# Genome-wide identification and comprehensive analysis of *EuFLS* genes in *Eucommia ulmoides* reveals their roles in growth, development, and abiotic stress response

**DOI:** 10.3389/fpls.2025.1662635

**Published:** 2025-10-23

**Authors:** Jun Liu, Tingting Cheng, Lili Wang, Conglong Lian, Rui Ma, Weimeng Feng, Jinxun Lan, Bao Zhang, Qingxin Du, Jiefeng Kou, Suiqing Chen

**Affiliations:** ^1^ School of Pharmacy, Henan University of Chinese Medicine, Zhengzhou, Henan, China; ^2^ Research Institute of Non-Timber Forestry, Chinese Academy of Forestry, Zhengzhou, China; ^3^ International Center for Bamboo and Rattan/Key Laboratory of Bamboo and Rattan Science and Technology, National Forestry and Grassland Administration, Beijing, China; ^4^ Changjiang Basin Ecology and Environment Monitoring and Scientific Research Center, Changjiang Basin Ecology and Environment Administration, Ministry of Ecology and Environment, Wuhan, China; ^5^ China Collaborative Innovation Center of Research and Development on the Whole Industry Chain of Yu-Yao, Zhengzhou, Henan, China

**Keywords:** *Eucommia ulmoides*, FLS gene family, genome-wide identification, growth and development, abiotic stress, expression pattern analysis

## Abstract

Flavonoids with great medicinal value play an important role in plant individual growth and stress resistance. Flavonol synthase (FLS) is one of the key enzymes to synthesize flavonoids. However, there is no information available about FLS family in *Eucommia ulmoides*, an ancient and precious plant with great economic value. In this study, twelve *EuFLS* genes were identified and classified into two distinct subgroups based on their phylogenetic trees, these genes were unevenly distributed across eight chromosomes. Gene structure analysis revealed that *EuFLS* genes contained between two and four introns. The number of introns within members of the same evolutionary branch was generally consistent. The *EuFLS* promoters region contained a substantial number of hormone-responsive, stress-responsive, and light-responsive. RNA-seq data revealed tissue-specific expression patterns, *EuFLS2* and *EuFLS9* displayed the highest expression levels in leaves, whereas *EuFLS4* the peak expression level in the xylem. The majority of *EuFLS* genes showed higher expression levels in red leaves and male flowers; furthermore, these genes contributed to leaf development and rubber biosynthesis. qRT-PCR analysis showed that most *EuFLS* genes downregulation under ABA and SA treatments. *EuFLSs* displayed divergent expression trends under MeJA treatment. While drought stress significantly induced the expression of most *EuFLSs*, especially, *EuFLS9* was induced 50-fold at 3 h, suggesting that *FLS* genes in *E. ulmoides* regulate plant growth and respond to different stresses by following different hormone signaling pathways, which laid a valuable foundation for further understanding the function of *FLS* genes in multiple stress responses and phytohormone crosstalk in *E. ulmoides*.

## 1 Introduction

*Eucommia ulmoides*, a member of the genus *Eucommia* within the family Eucommiaceae, is a deciduous tree characterized by its gray-brown bark and is a dioecious plant with unisexual flowers occurring on separate individuals (Editorial Committee of Flora of China and Chinese Academy of Sciences, 1979). *E. ulmoides* demonstrates remarkable adaptability and is primarily distributed across Guizhou, northern Sichuan, southern Shaanxi, western Henan, western Hubei, and western Hunan. Recognized as a rare and endangered medicinal plant endemic to China, *E. ulmoides* has been extensively utilized in traditional Chinese medicine for centuries. It is listed as a superior-grade herb in the “Shennong Bencao Jing”. Flavonoids are one of the main active ingredients in *E. ulmoides*, and they possess various pharmacological properties, such as lowering blood sugar, blood pressure, blood lipids, antioxidation, and enhancing immunity (Gong et al., 2022; Li et al., 2022; Li et al., 2024).

Flavonoids, important secondary metabolites, are extensively distributed in the plant kingdom and can be divided into six categories including flavones, isoflavones, flavanones, flavonols, flavanols, and anthocyanins (Harborne and Williams, 2000; Winkel-Shirley, 2002; Martens et al., 2010; Wang et al., 2021b). These compounds are characterized by their low molecular weight and wide distribution. They contribute to the formation of plant leaf color, flower color, fruit color, and seed color and also play significant roles in plant growth and development, mediating responses to abiotic stresses and enhancing resistance against pathogen infections. Moreover, flavonoids are actively involved in the complex signaling networks that govern plant-microbe interactions, facilitating the transmission of relevant biochemical signals (Schijlen et al., 2004). Additionally, they serve as a protective barrier, shielding plant tissues from the detrimental effects of ultraviolet (UV) radiation (Stracke et al., 2010). In *E. ulmoides*, the accumulation of most flavonoids peak in growing leaves, follow by old leaves, and the expression levels of most *F3’H*, *FLS*, *CHI* and *CHS* isoforms are relatively high in growing leaves, indicating that isoforms in the flavonoid biosynthetic pathway and flavonoid metabolic pathway are highly expressed in growing leaves and old leaves (Li et al., 2019).

Flavonol synthase (FLS) is a key enzyme in synthesis of flavonols, which can control the flux of flavonoids into the branch pathway of flavonols synthesis to form various flavonol compounds which are present mainly in the form of quercetin and kaempferol (Winkel-Shirley, 2002; Falcone Ferreyra et al., 2012). FLS belongs to the 2-oxoglutarate-dependent dioxygenase (2-ODD) family of non-heme iron enzymes characterized by two conserved functional domains: The N-terminal dioxygenase domain (DIOX_N) and the C-terminal 2-oxoglutarate/Fe(II)-dependent oxygenase domain (2OG-Fe II_Oxy) (Huang and Cai, 2014; Shi et al., 2019). The enzymatic activity of FLS was first identified in petunias (Forkmann et al., 1986). *FLS* genes have been then identified in various organisms. The tobacco genome contains nine *NtFLS* genes, with *NtFLS1* and *NtFLS2* significantly regulated by various hormones and abiotic stressors (Shi et al., 2017). In *Brassica napus*, 13 *FLS* genes have been characterized, among which *BnaFLS1–1* and *BnaFLS1–2* are associated with flavonol accumulation (Schilbert et al., 2021). Furthermore, the overexpression of the *AvFLS* gene increases the total flavonoid content of tobacco and enhances antioxidant enzyme activity (Wang et al., 2021a). In *Brassica vegetables*, the silencing of *BrFLS1* reduces plant resistance to low temperatures (Yue et al., 2024). In alfalfa, cold stress triggers the expression of the *MsFLS* gene family, with *MsFLS7*, *MsFLS9*, *MsFLS10*, *MsFLS11*, *MsFLS13*, *MsFLS16*, *MsFLS17* and *MsFLS18* showing significant upregulation, and the overexpression of *MsFLS13* significantly improve cold stress tolerance and antioxidant capacity and reduced membrane oxidative damage (Zhang et al., 2025). In safflower, the expression level of *CtFLS1* show a positive correlation with the accumulation level of total flavonoid content, *CtFLS1*-overexpression *Arabidopsis* plants significantly induce the expression levels of key genes involved in flavonol pathway, promoted seed germination, as well as resistance to osmotic pressure and drought, and reduced sensitivity to ABA compared to mutant and wild-type plants (Ma et al., 2024). In *Juglans sigillata, JsFLS5* overexpression in calli effectively mitigates the oxidative damage induced by osmotic stress and reduces Na^+^ toxicity by enhancing flavonoid synthesis under salt stress conditions (Wei et al., 2023). In apples, overexpression of *dihydroflavonol 4-reductase* (*DFR*) or the silencing of the *FLS* gene results in a significant increase in anthocyanin accumulation in leaves, leading to the formation of red-leaf phenotypes (Tian et al., 2015). Similarly, during the ripening of jujube fruits, the deepening of fruit peel coloration is associated with *FLS* downregulation and a concomitant *DFR* upregulation (Wang, 2021). This regulatory dynamic is further supported by studies in tobacco, where *FLS*-silenced lines exhibit a marked reduction in flavonol content and a corresponding increase in anthocyanin levels (Mahajan et al., 2012). In addition to their role in pigmentation, flavonols synthesized by the action of FLS exert protective effects against ultraviolet (UV) radiation damage in both apple (Solovchenko and Schmitz-Eiberger, 2003) and maize (Falcone Ferreyra et al., 2010). Notably, under reduced UV irradiation and prolonged transcriptional activity of key regulatory factors, such as HYPOCOTYL5 (HY5), MYB10, and MYB22, *FLS* is downregulated in apple throughout the fruit development process (Henry-Kirk et al., 2018).

Research has shown that heterologous expression of *Apocynum venetum FLS* in *Arabidopsis thaliana* significantly enhanced flavonoid accumulation and photosynthetic efficiency while simultaneously reducing malondialdehyde (MDA) content. These changes collectively contribute to a marked improvement in the salt stress tolerance of transgenic *Arabidopsis* (Cai, 2022). Furthermore, studies have demonstrated that various environmental and chemical stimuli, including ultraviolet (UV) radiation, sodium chloride (NaCl) stress, and exogenous application of abscisic acid (ABA) and salicylic acid (SA), can upregulate the expression of *Ginkgo biloba FLS*. This upregulation leads to increase flavonol biosynthesis, thereby enhancing the plant’s ability to withstand abiotic stresses (Xu et al., 2011). Similarly, in tobacco, the heterologous expression of *Muscari armeniacum MaFLS* result in a notable decrease in anthocyanin content in the corolla of transgenic tobacco, accompanied by a significant increase in total flavonol levels. Quantitative real-time-polymerase chain reaction (qRT-PCR) analysis reveal that the expression levels of key anthocyanin biosynthesis-related genes, including *NtCHS*, *NtF3H*, *NtDFR*, *NtANS*, and the R2R3MYB transcription factor (*NtAN2*), are significantly downregulated in transgenic tobacco. Conversely, the *NtFLS* gene is significantly upregulated, leading to corolla coloration ranging from light pink to nearly white (Liu, 2019). Since the discovery of the *FLS* gene in *Petunia* in 1993 (Kim et al., 2014), *FLSs* have been successfully cloned and characterized in a wide range of species, including *Populus deltoides* (Kim et al., 2010), *Zea mays* (Cai, 2022), *G. biloba* (Xu et al., 2011), *Musa nana* (Xiao et al., 2024), *Dioscorea esculenta* (Kou et al., 2023), and *Medicago sativa* (Zhang et al., 2023). These findings highlight the conserved yet versatile role of *FLSs* in regulating flavonoid metabolism across diverse plant species.

Although *FLSs* have been extensively studied in a wide range of plant species, the *FLS* gene family in *E. ulmoides* remains unexplored. Based on the genomic data of *E. ulmoides*, this study employed comprehensive bioinformatics approaches to systematically identify and characterize members of its FLS gene family. Furthermore, the transcriptomic data was utilized to investigate the potential roles of *EuFLS* genes during different developmental stages, in the context of leaf color variations, and in the regulation of rubber biosynthesis in *E. ulmoides*. The expression characteristics of *EuFLS*s in various tissues and developmental stages, as well as their expression responses to different hormonal treatments and drought stress were analyzed using qRT-PCR. Collectively, the research provides a robust theoretical foundation for elucidating the functional roles of *EuFLSs* in *E. ulmoides*.

## 2 Materials and methods

### 2.1 Identification of *EuFLS* genes in *E. ulmoides*

The FLS protein sequences of Arabidopsis and rice were obtained from The Arabidopsis Information Resource (https://www.arabidopsis.org/) and Rice Data (http://www.ricedata.cn/gene/), respectively. The protein sequence of *E. ulmoides* was obtained from the its genome database with the accession number PRJNA599775; GCA_016647705.1 in the NCBI database (https://www.ncbi.nlm.nih.gov/). To identify non-redundant *FLS* genes in the *E. ulmoides* genome, we employed the Hidden Markov Model (HMM) profile of the 2OG-FeII_Oxy (PF03171) and DIOX_N (PF14226) with an e-value threshold of 10^-5^. Redundant sequences were excluded from subsequent analyses based on sequences alignment results unsing Cluster W (Tompson et al., 1994). To confirm the FLS domain in identified proteins, domain analysis was performed using InterProScan tool (http://www.ebi.ac.uk/Tools/pfa/iprscan5/) (Quevillon et al., 2005). The candidate sequences were further validated using three online tools: NCBI Conserved Domain Search Service (CD Search; http://forestry.fafu.edu.cn/db/PhePacBio/blast/blast_cs.php), Pfam (http://pfam.sanger.ac.uk/), and SMART (http://smart.embl-heidelberg.de/) (Letunic et al., 2009), to confirm the presence of the DIOX_N domain, which is unique to the FLS gene family. Physicochemical parameters, including molecular weight (MW) and isoelectric point (pI), of each EuFLS protein were predicted using the pI/Mw tool from ExPASy (http://www.expasy.org/tools/) with the resolution parameter set to “average” (Gasteiger et al., 2003). Three-dimensional (3D) structures of EuFLS proteins were modeled using SWISS-MODEL (https://swissmodel.expasy.org/) (Waterhouse et al., 2018).

### 2.2 Phylogenetic analysis, gene structural, and conserved motif of FLS in *E. ulmoides*

A total of 12 FLS protein sequences from *E. ulmoides* were aligned using Clustal W with default parameters, An unrooted phylogenetic tree was subsequently constructed using MEGA6.0 software via the Neighbor-joining (NJ) algorithm, and the support of the tree topology was assessed by bootstrap analysis with 1000 replicates (Tamura et al., 2013). For comparative phylogenetic analysis, a neighbor-joining phylogenetic tree was constructed using FLS protein sequences from multiple species, including *Arabidopsis*, *Arabidopsis lyrata*, *Arabidopsis halleri*, *Antirrhinum majus*, *Arabis nemorensis*, *Allium cepa*, *Camellia nitidissima*, *Cyclamen purpurascens*, *Camellia sinensis*, *Capsella rubella*, *Citrus unshiu*, *Capsella grandiflora*, *Brassica oleracea*, *Brassica napus*, *Brassica rapa*, *Brassica cretica*, *Boechera stricta*, *Eustoma russellianum*, *Eutrema salsugineum*, *Zea mays*, *Fagopyrum dibotrys*, *Fagopyrum esculentum*, *Fagopyrum tataricum*, *Fragaria x ananassa*, *Petroselinum crispum*, *Ginkgo biloba*, *Gentiana triflora*, *Glycine max*, *Muscari aucheri*, *Malus domestica*, *Rosa rugosa*, *Microthlaspi erraticum*, *Narcissus tazetta*, *Vaccinium corymbosum*, *Prunus persica*, *Vitis vinifera*, *Petunia x hybrida.*

To analyze the exon-intron organization of the *EuFLS* genes, the complete *E. ulmoides* genome and coding sequences were obtained from the Genome Warehouse Database. The Gene Structure Display Server (GSDS) was employed to visualize the exon-intron structures of individual *EuFLS* genes by aligning their cDNAs sequences with the corresponding genomic DNA sequences (Guo et al., 2007). To identify conserved motifs within EuFLS proteins, the online MEME (Multiple Expectation Maximization for Motif Elicitation) program (http://memesuite.org/tools/meme) was used with the following parameters: number of repetitions = any, maximum number of motifs = 20, minimum width ≥ 6, maximum width ≤ 200. Only motifs with an E-value < 1e-20 were retained for further analysis (Bailey et al., 2006).

### 2.3 Chromosomal distribution, and cis-regulatory element analysis in the promoter regions of *FLS* genes in *E. ulmoides*

The chromosomal locations of *EuFLS* genes were determined using TBtools (Chen et al., 2023) based on the genomic data of *E. ulmoides*. To identify the cis-elements in the promoter region of each *FLS* gene in *E. ulmoides*, the 2000 bp upstream sequences of the *FLS*-encoding DNA sequences were extracted from the available *E. ulmoides* genome data using the TBtools program, and possible cis-acting elements were analyzed using the PlantCARE database (http://bioinformatics.psb.ugent.be/webtools/plantcare/index.html). The cis-element distributions were visualized using TBtools.

To investigate the association between EuFLS genes and genes involved in the *E. ulmoides* rubber biosynthesis pathway, we constructed a co-expression network using the values of these target genes. Pearson’s correlation analysis was conducted to support the network construction, utilizing OmicStudio tools (https://www.omicstudio.cn/tool/62).

### 2.4 Expression patterns of *EuFLSs* in different tissues and different developmental stages based on the public RNA-seq data sets

Transcriptome data of *E. ulmoides* across different leaf developmental stages (leaf buds, growing leaves, young leaves and mature leaves) were obtained from the NCBI Sequence Read Archive (SRA) database was SRP218063 (Li et al., 2019). Different tissues (leaves, seeds, marginal pericarp, central pericarp, and xylem) with SRA accessions SRX7525252-54, SRX7532003-05, SRX7531725-27, and SRX7533248-50, leaves of different colors (green leaves and purple leaves) under accession SRP327902. Different developmental stages of floral with accessions SRR2170964 and SRR2170970) (Qing et al., 2021). In addition, RNA-seq data from two *E. ulmoides* cultivars, ‘Xiaoye’ and ‘Qinzhong 2’ under accessions SRP158357 (Ye, 2019). these datasets were used to investigate the expression patterns of EuFLS genes during *E. ulmoides* rubber biosynthesis. The expression abundance of EuFLS genes was determined using their Fragments Per Kilobase of transcript per Million mapped reads (FPKM) values. Notably, some EuFLS genes exhibited an FPKM value of 0, which likely reflects expression levels below the detection limit of the current transcriptomic sequencing technology rather than absolute absence of expression. A heatmap of EuFLS*s* expression was generated using TBtools, with expression values represented as log_2_(FPKM) to standardize the data.

### 2.5 Plant materials and stress treatments

The *E. ulmoides* seedling were cultured in a growth chamber with a 16-h light/8-h dark cycle at 22°C/18°C, 80% humidity, and watered with Hoagland nutrient solution every week. In the drought stress treatment, a 20% PEG6000 solution was poured into the medium vermiculite with *E. ulmoides* seedlings. To examine the effect of hormones on the expression of *EuFLS* genes, 2-month-old seedling leaves of *E. ulmoides* were sprayed with 200 mM Gibberellic acid (GA_3_), ABA, Methyl Jasmonate (MeJA) and SA, until there was liquid dripping. Untreated samples were used as the control treatment. The leaves were sampled at 0, 6, 12, 24, 36, 48, and 72 h after hormone treatments, frozen in liquid nitrogen, and finally stored at -80°C for RNA extraction. There were three independent replicates for each treatment.

### 2.6 RNA extraction, cDNA synthesis, and qRT-PCR gene expression analysis

The RNA extraction kit (TIANGEN Biotechnologies, DP441, China) was used to extract total RNA according to the manufacturer’s instructions. A NanoDrop ND-2000 (Thermo Scientific, USA) spectrophotometer and 1% agarose gel electrophoresis were used to detect the RNA quality of all samples. For qRT-PCR, Primer 3 web (http://primer3.ut.ee/) was used to design the specific primers based on the coding sequence of *EuFLS* genes. The primers of all the *EuFLS* genes were listed in **Supplementary Table S1**. The *Ubiquitin-conjugating enzyme S* (*UBC*) was used as the internal reference gene (Li et al., 2020b). The qRT-PCR reactions were conducted on a Quant Studio 5 (ABI, USA) using the 2×Universal SYBR Green Fast qPCP Mix. The PCR reaction mixture (20 µL) contained 7.2 µL of cDNA, 10 µL of SYBR Advantage Premix, 0.4 µL of each primer (10 nmol mL^-1^). The following amplification reactions were performed for qRT-PCR: 95° C for 5 s, 60°C for 30 s, 40 cycles. The ΔCT and ΔΔCT values were calculated using the 2^-ΔΔCT^ method. To ensure the accuracy of the data, we performed 3 biological replicates and 3 technical replicates.

## 3 Results

### 3.1 Identification and characterization of *EuFLS* gene family members

Using the *AtFLS* sequence as a reference and performing comparative analyses with the NCBI and Pfam databases, a total of 12 *EuFLSs* were identified in the *E. ulmoides* genome, designated as *EuFLS1* to *EuFLS12*. The physicochemical properties of the EuFLSs were analyzed using the ExPASy tool. The results showed that the polypeptides encoded by the 12 *EuFLSs* comprised 328–420 amino acids (aa) (**Table 1**), with the longest being EuFLS9 (420 aa) and the shortest being EuFLS7 (328 aa). The molecular weight (MW) of the proteins varied from 36297.19 Da (EuFLS7) to 46513.98 Da (EuFLS9), and the isoelectric point (pI) ranged from 5.46 (EuFLS1) to 8.72 (EuFLS10). Notably, only EuFLS8 and EuFLS10 exhibited pI values >7, classifying them as basic proteins. The remaining EuFLSs had pI values <7, indicating that they were acidic in nature. Regarding protein stability, the instability indices of the EuFLSs ranged from 32.70 (EuFLS4 and EuFLS5) to 51.45 (EuFLS8). The instability indices of EuFLS3, EuFLS4, EuFLS5, and EuFLS7 below 40, categorizing them as stable proteins. The remaining EuFLSs were classified as unstable.

**Table 1:** Analysis of physical and chemical properties of FLS proteins from *E. ulmoides*.

Gene ID	Gene name	Number of amino acids	Molecular weigh	Theoretical pI	Instability index	Aliphatic index	Grand average of hydropathicity
IMPGEUL1N42151	*EuFLS1*	359	40565.32	5.46	40.20	87.94	-0.361
IMPGEUL1N10568	*EuFLS2*	336	38030.59	5.59	41.95	89.58	-0.412
IMPGEUL1N17905	*EuFLS3*	348	40378.58	5.61	35.87	76.95	-0.630
IMPGEUL1N18731	*EuFLS4*	348	39991.46	5.94	32.70	81.44	-0.532
IMPGEUL1N4022	*EuFLS5*	352	40507.59	5.76	32.70	71.16	-0.618
IMPGEUL1N2699	*EuFLS6*	337	38665.88	5.65	47.33	85.82	-0.375
IMPGEUL1N104453	*EuFLS7*	328	36297.19	6.19	35.92	84.05	-0.263
IMPGEUL1N76927	*EuFLS8*	347	38205.63	7.13	51.45	81.21	-0.182
IMPGEUL1N45050	*EuFLS9*	420	46513.98	5.63	50.95	82.21	-0.433
IMPGEUL1N62711	*EuFLS10*	357	40327.19	8.72	45.19	85.15	-0.386
IMPGEUL1N102331	*EuFLS11*	346	39135.45	6.45	41.01	90.40	-0.330
IMPGEUL1N60477	*EuFLS12*	351	39687.31	5.82	45.25	88.83	-0.372

**Figure 1:**
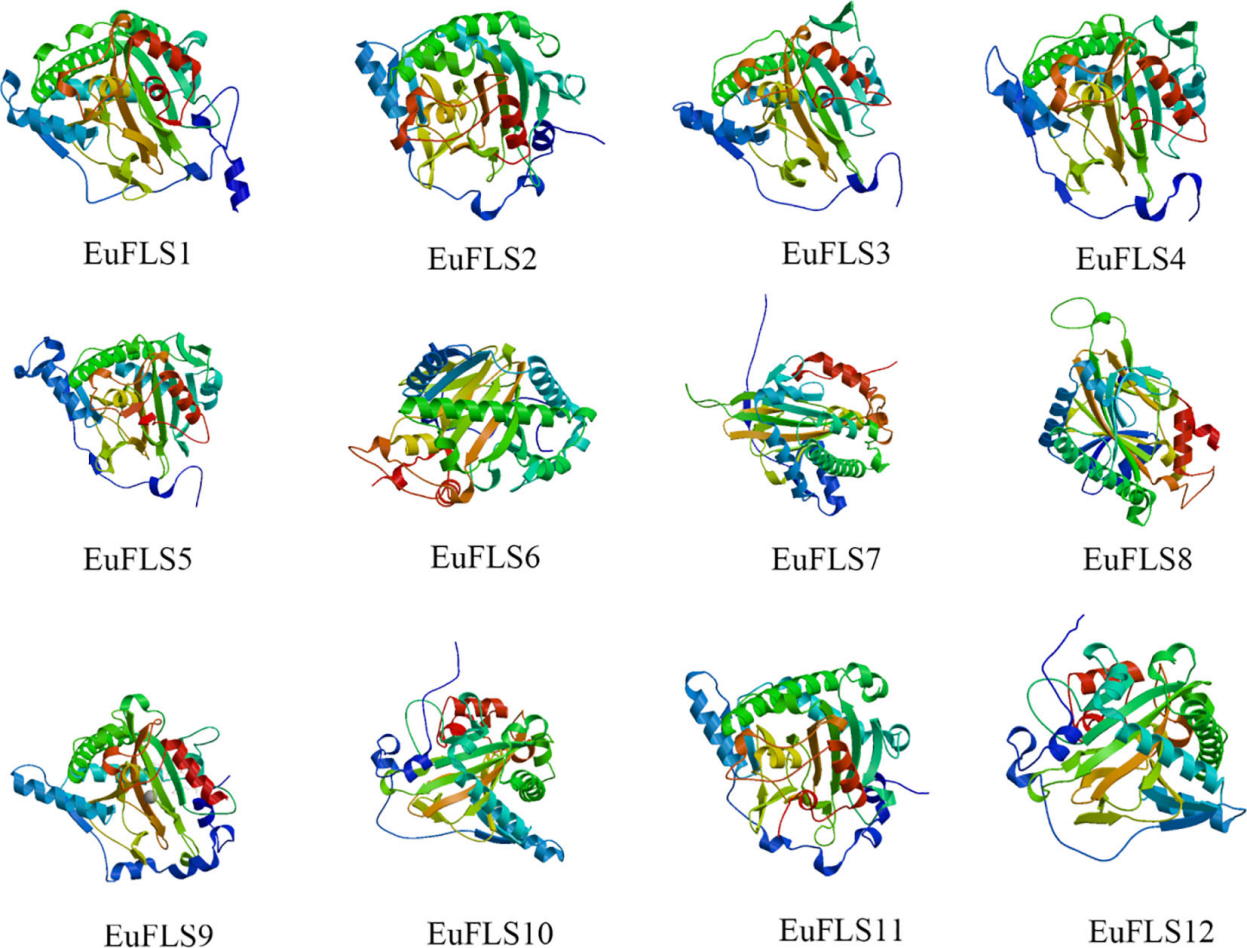
The tertiary structure prediction of EuFLS proteins in *E. ulmoides*.

The aliphatic indices of EuFLSs ranged from 87.40 (EuFLS5) to 131.81 (EuFLS7), while the grand average of hydropathicity (GRAVY) values varied between −0.412 (EuFLS2) and 0.263 (EuFLS7). Among the 12 EuFLSs, six proteins exhibited GRAVY values of <0, indicating that they were hydrophilic in nature, while the remaining proteins were classified as lipophilic. A secondary structure analysis demonstrated that all proteins contained α-helices, extended chains, and random coils; however, no β-turns were detected (**Supplementary Table S2**). Specifically, the proportions of α-helices and random coils ranged from 32.12% to 41.71% and 49.86% and 59.59%, corresponding to aa counts ranging from 62 to 320 aa and 104 to 406 aa, respectively. Overall, the secondary structure composition of the 12 EuFLSs was in the following order based on abundance: random coils > α-helices > extended chains > β-turns. Furthermore, to obtain more structural insights, the 3D structures of EuFLS proteins were also modeled. All these 3D structures had an almost similar structural composition with secondary structures (**Figure 1**).

### 3.2 Phylogeny and classification of EuFLSs

**Figure 2:**
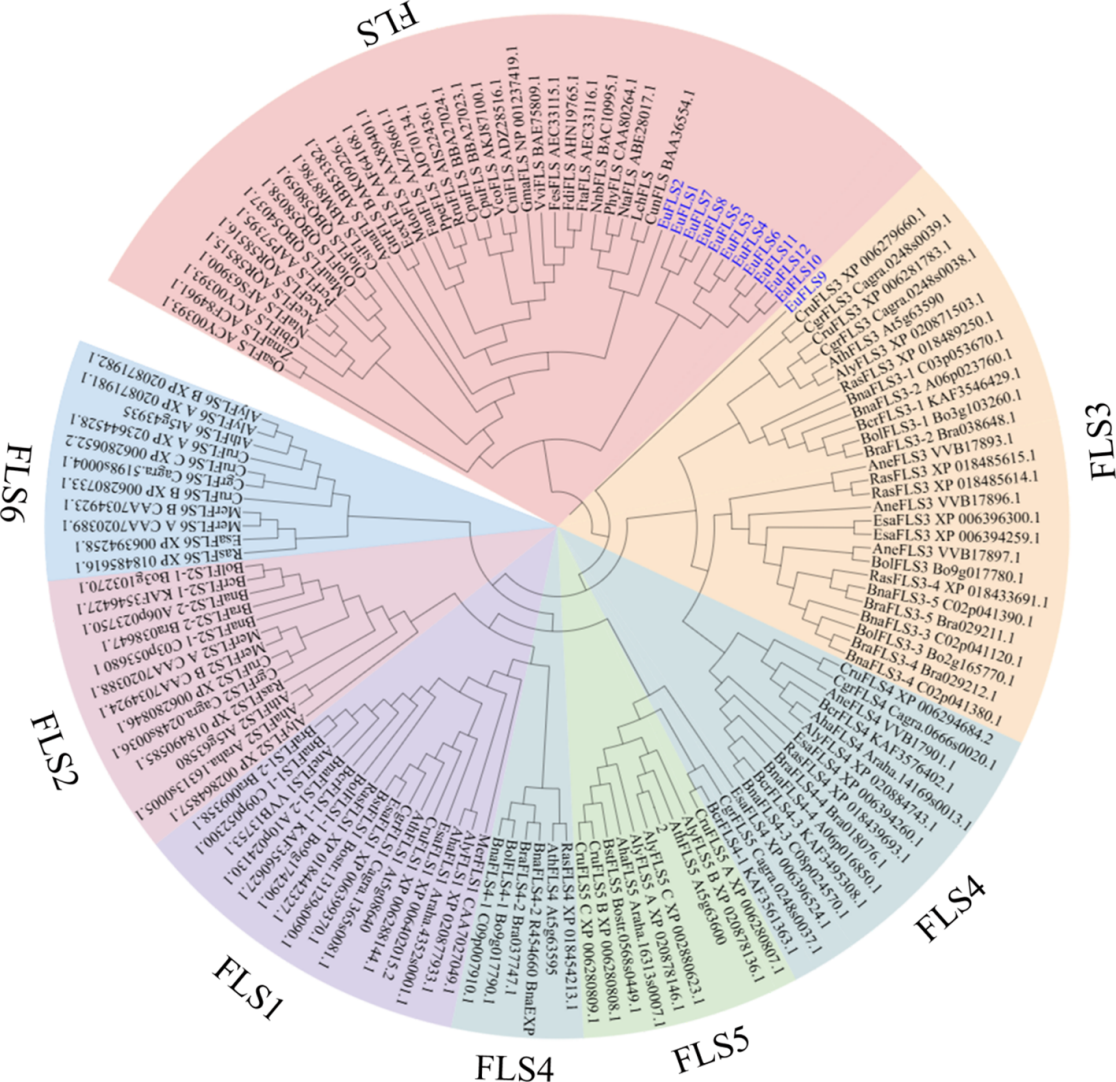
Phylogenetic tree of FLS protein from E. ulmoides, Arabidopsis (AthFLS), Arabidopsis lyrata (AlyFLS), Arabidopsis halleri (AhaFLS), Antirrhinum majus (AmaFLS), Arabis nemorensis (AneFLS), Allium cepa (AceFLS), Camellia nitidissima (CniFLS), Cyclamen purpurascens (CpuFLS), Camellia sinensis (CsiFLS), Capsella rubella (CruFLS), Citrus unshiu (CunFLS), Capsella grandiflora (CgrFLS), Brassica oleracea (BolFLS), Brassica napus (BnaFLS), Brassica rapa (BraFLS), Brassica cretica (BcrFLS), Boechera stricta (BstFLS), Eustoma russellianum (EexFLS), Eutrema salsugineum (EsaFLS), Zea mays (ZmaFLS), Fagopyrum dibotrys (FdiFLS), Fagopyrum esculentum (FesFLS), Fagopyrum tataricum (FtaFLS), Fragaria x ananassa (FanFLS), Petroselinum crispum (PcrFLS), Ginkgo biloba (OsaFLS), Gentiana triflora (GtrFLS), Glycine max (GmaFLS), Muscari aucheri (MauFLS), Malus domestica (MdoFLS), Rosa rugosa (RruFLS), Microthlaspi erraticum (MerFLS), Narcissus tazetta (NtaFLS), Vaccinium corymbosum (VcoFLS), Prunus persica (PpeFLS), Vitis vinifera (VviFLS), Petunia x hybrida (PhyFLS), EuFLS proteins were marked in blue.

To elucidate the evolutionary relationships among EuFLSs, a neighbor-joining phylogenetic tree was constructed using 139 FLS protein sequences from 38 plant species. The EuFLS candidates were further classified within the FLS gene family based on their phylogenetic relationship to their most likely *Arabidopsis* orthologs. The result showed that 139 FLSs were classified into seven distinct subfamilies: FLS, FLS1-FLS6 (**Figure 2**). Among these, Group FLS was the largest, with 43 FLSs, while Group FLS5 comprised 10 FLSs, which was the smallest subfamilies. EuFLSs were distributed across Groups FLS and FLS3. Specifically, Group FLS comprised the majority of EuFLSs (n = 11, 91.67%), while Group FLS3 contained only one EuFLS (EuFLS9).

### 3.3 Gene structures, protein conserved domain, and motif analysis of EuFLSs

**Figure 3:**
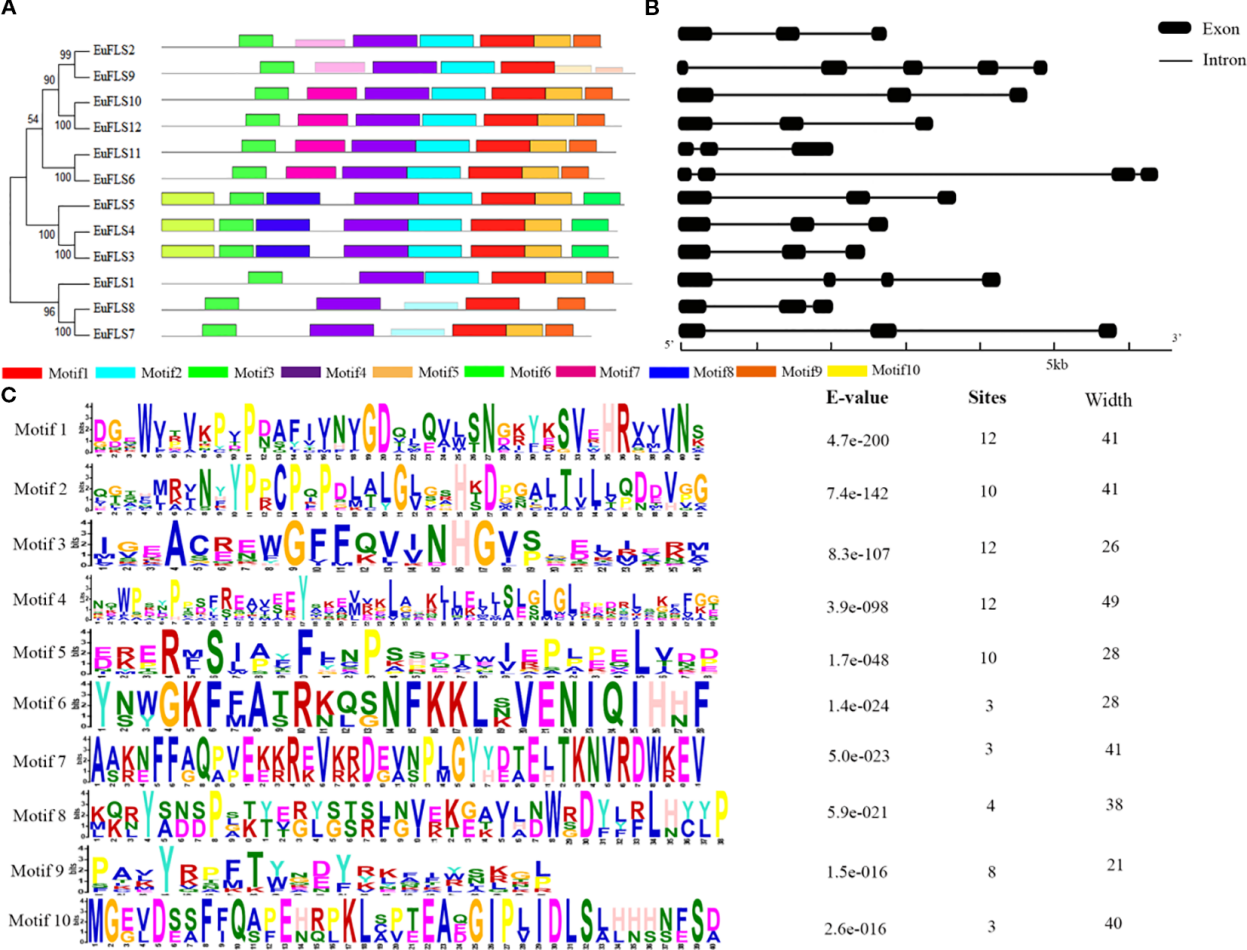
Analysis of conserved motifs and gene structures of EuFLS proteins. **(A)** Motif distribution of *EuFLSs* members. **(B)** Exon-intron structure analysis of *EuFLSs*. Black boxes, and black lines represent exons and introns, respectively. **(C)** Conserved motif amino acid sequence of *EuFLSs* members.

To gain deeper insights into the structural characteristics of the *EuFLS* gene family in *E. ulmoides*, we conducted a comprehensive analysis of the conserved motifs and intron-exon structures in these genes. A total of ten conserved motifs were identified and designated as Motifs 1-10. The results showed that with the exception of EuFLS8, all other EuFLSs contained Motifs 1-5 (**Figure 3A**). Notably, Motif 6, Motif 8 and Motif 10 domain were exclusively present in EuFLS3, EuFLS4 and EuFLS5, however these proteins did not contain Motif 9. Furthermore, Motif 7 domain was exclusively present in (EuFLS2/6/9/10/11/12), suggesting that the functional divergence of *EuFLSs* might be closely associated with their motif compositions.

Gene structure analysis showed that the number of exons in the *EuFLS*s ranged from 3 to 5 (**Figure 3B**), *EuFLS1*, and *EuFLS6* contained four exons each, only *EuFLS9* comprised five exons. The protein evolutionary tree of the EuFLSs indicated that the structures of the proteins in the same subgroup were similar (**Figure 3B**). Overall, the *EuFLS*s structures were relatively simple, and most of them had 2–3 exons. We speculated that the *EuFLS*s may have been produced or differentiated relatively late, and that their functions are relatively specific. The proteins that were encoded by genes with similar exon and intron structures also had high homology in the phylogenetic tree, indicating that closely related genes shared similar exon/intron structures in their evolution. The lengths of *EuFLSs* intron and exon locations and numbers were different among the different members of the *EuFLSs* gene family, indicating that the *EuFLSs* family had undergone strong differentiation during their evolution. The diversity of the intron/exon structure of the *EuFLSs* and the relative consistency within each subgroup indicated the *EuFLSs* structures were conserved within a subgroup and the diversity in evolution.

### 3.4 Chromosomal distribution, and cis-regulatory element analysis in the promoter regions of *FLS* genes in *E. ulmoides*

**Figure 4:**
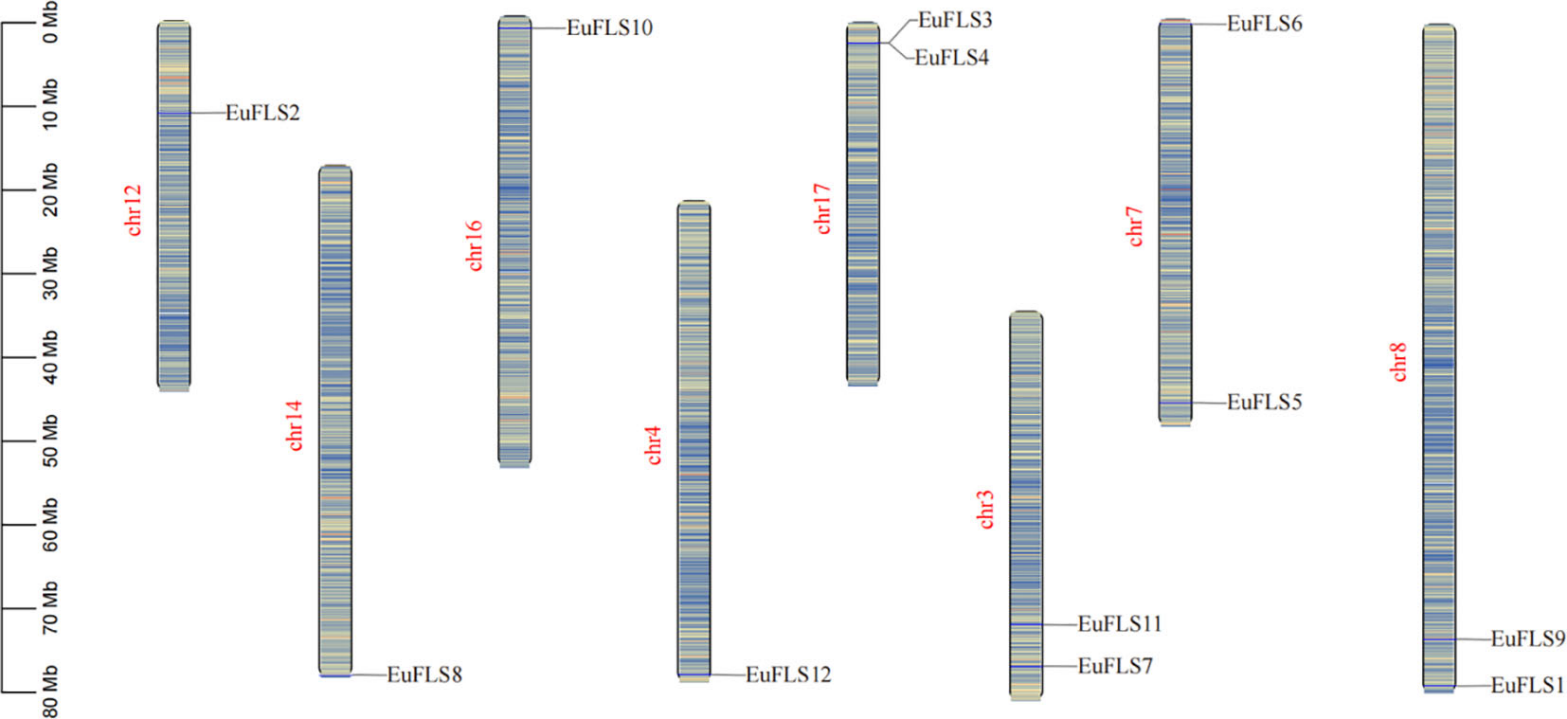
Chromosomal distribution of *FLF* genes in *E. ulmoides.* The scale is in megabases (Mb), with chromosome lengths indicated on the left side.

Based on the annotated genome information of *E. ulmoides*, the chromosomal localization of *EuFLSs* was determined. Our results revealed that the 12 *EuFLSs* were unevenly distributed across eight *E. ulmoides* chromosomes (**Figure 4**). Notably, chromosome 3/7/8/17 harbored two members. Each of the remaining chromosomes carried only a single *EuFLS*. This uneven distribution pattern suggested potential functional or evolutionary significance associated with the clustering of *EuFLSs* on specific chromosomes.

**Figure 5:**
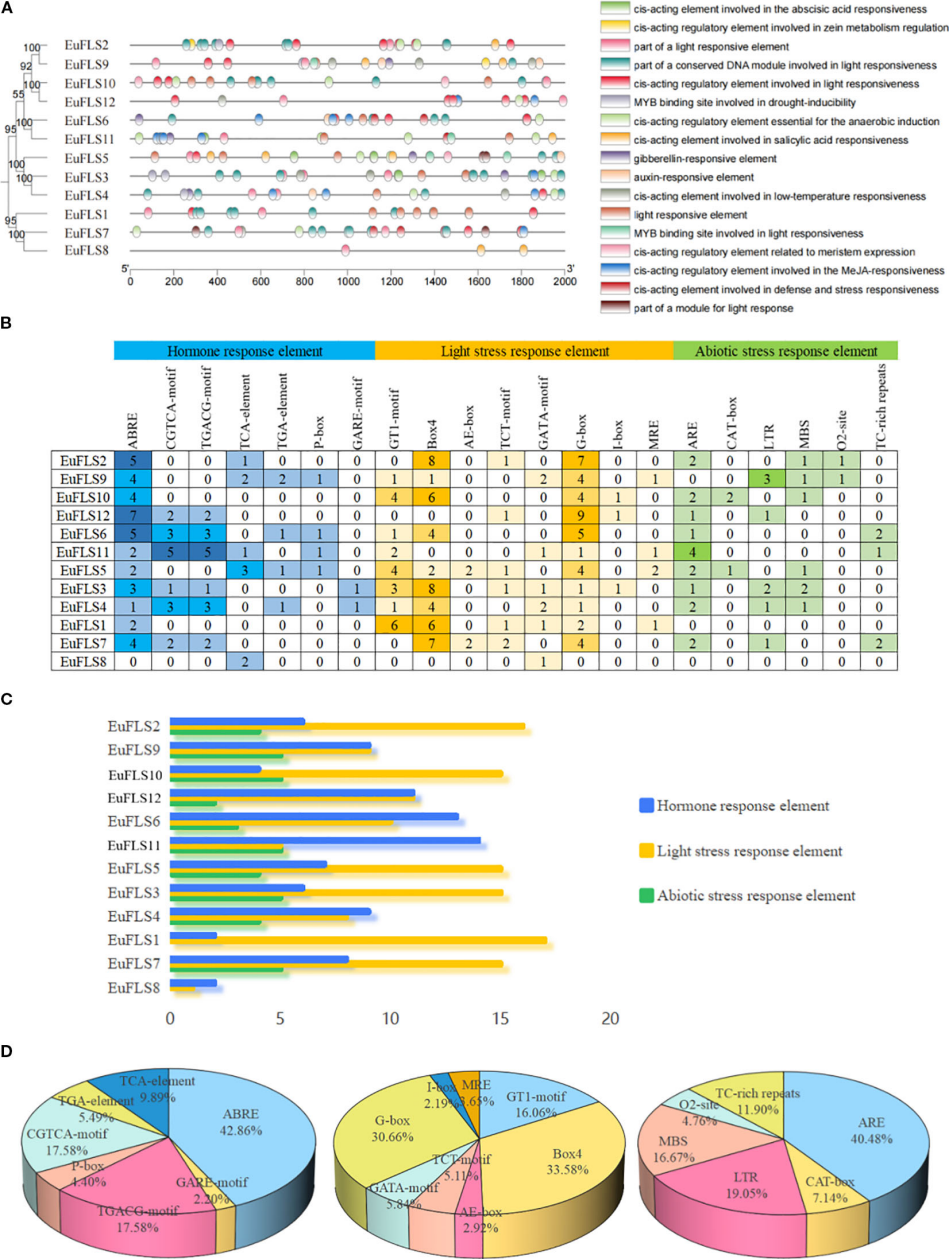
Analysis of promoter elements of *EuFLS* genes. **(A)** location map of promoter elements of *EuFLSs* genes. **(B)** number map of each elements in *EuFLSs* genes, the gradient colors in the grid correspond to the quantity of cis-acting elements in *EuFLSs*. **(C)** total number map of each type of elements in *EuFLSs* genes. The multicolored histogram indicates the cis-elements in each category. These elements are divided into three categories in terms of the functional notes: plant hormone response element, light stress response element, and abiotic stress response element. **(D)** proportion map of each element in three categories of elements (from left to right are hormone response element, light stress response element, abiotic stress response element).

The 2000-bp upstream promoter regions of the 12 *EuFLSs* were analyzed for cis-acting elements (**Figure 5**). A total of 270 cis-acting elements were identified, which were categorized into three functional groups: Hormone- (ABRE, CGTCA-motif, TGACG-motif, TCA-element, TGA-element, P-box, GARE-motif, etc.), light- (GT1-motif, Box4, AE-box, TCT-motif, GATA-motif, G-box, I-box, MRE, etc.), and abiotic stress-responsive elements (ARE, CAT-box, LTR, MBS, 02-site, TC-rich repeats, etc.). Among these, 91, 137, and 42 elements were hormone-, light-, and abiotic stress-responsive elements. Among the light-responsive elements, the Box 4 element was the most abundant (46 elements, 33.58%), followed by G-box (42 elements, 30.66%), and GT1-motif (22 elements, 16.66%). ABRE element was highly abundant (39 elements, 42.68%) among the hormone-responsive elements, while TGACG-motif and CGTCA-motif were equally abundant (both 16 elements, 17.58%). Among abiotic stress-responsive elements, the ARE element was the most prevalent, accounting for 17elements (40.48%) in the abiotic stress-responsive elements. This was followed by the LTR element (8 elements, 19.05%), MBS element (7 elements, 16.67%) and the TC-rich repeats element (5 elements, 11.90%). These findings suggested that *EuFLSs* are involved in various biological processes in *E. ulmoides*, including growth and development, gibberellin signaling, photoperiod regulation, and responses to ABA, SA, anaerobic, and low-temperature stresses.

### 3.5 Analysis of expression patterns of *EuFLSs* at different developmental stages and with respect to *E.ulmoides* rubber content in leaves

**Figure 6:**
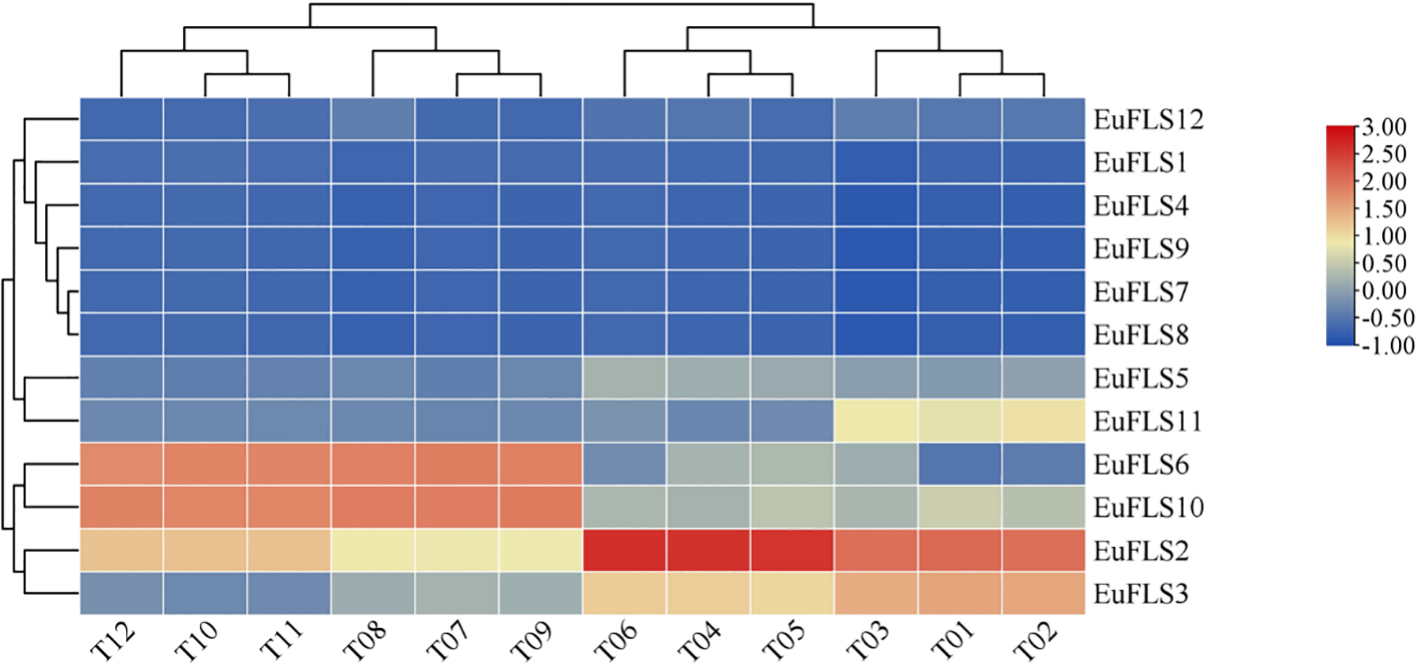
Expression patterns of *E. ulmoides* FLS gene family in different developmental stages of leaves. T01~T03: leaf buds; T04~T06: growing leaves; T07~T09: young leaves; T10~T12: mature leaves. The color key on the right represents the relative expression level in FPKM values, the res and blue bars (FPKM) indicate high and low expression, respectively.

Transcriptomic data from the leaves of *E. ulmoides* at different developmental stages (leaf buds: rudimentary stems with many spires; growing leaves: 3 cm in length; young leaves: new, fully expanded leaves; and mature leaves: 60 days after full expansion) were utilized to investigate the expression patterns of *EuFLSs*. As depicted in **Figure 6**, the FPKM values of *EuFLS7*, *EuFLS8*, and *EuFLS9* were consistently zero across all four-leaf developmental stages, indicating that these genes are not involved in leaf growth and development. Similarly, the FPKM value of *EuFLS4* was zero during the growing leaves, suggesting its lack of involvement at this stage. In contrast, *EuFLS2* exhibited the highest expression level in primary leaves, implying that it is mainly involved in this developmental stage. On the other hand, *EuFLS6* and *EuFLS10* exhibited the lowest transcription levels in leaf buds, with their expression levels gradually increasing as leaves matured. Their expression levels peaked in mature leaves, suggesting that *EuFLS6* and *EuFLS10* may play significant roles in the later stages of leaf development. These findings underscored the stage-specific functional roles of *EuFLSs* during leaf development in *E. ulmoides*, providing insights into their regulatory contributions at different developmental phases, laying the theoretical foundation for the subsequent functional studies of *EuFLSs* in the development of *E. ulmoides* leaves.

**Figure 7:**
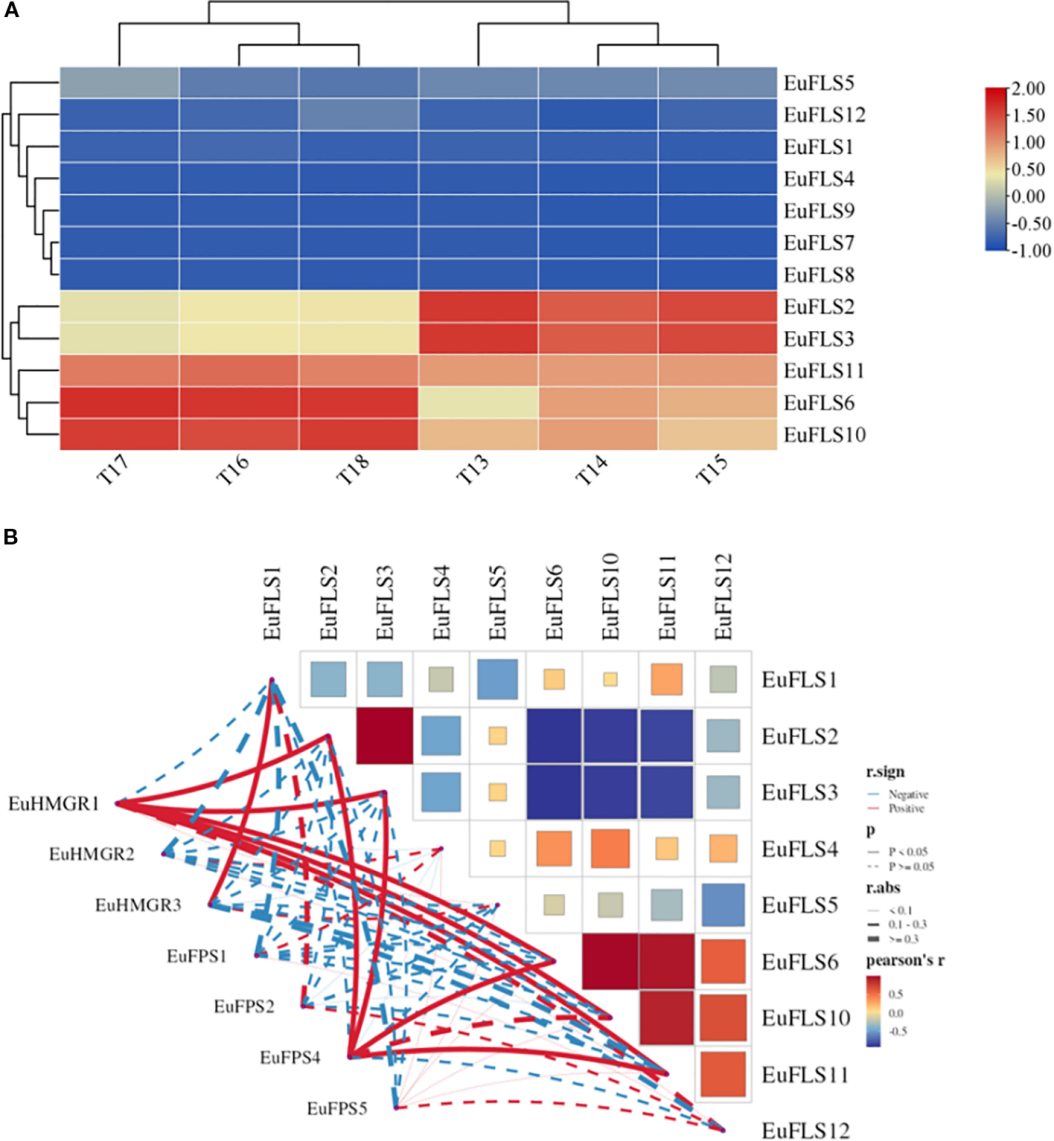
Expression patterns of *EuFLSs* in leaves with different *E*. *ulmoides* rubber content **(A)** and correlation analyses between *EuFLS* genes and *E*. *ulmoides* rubber biosynthesis genes **(B)**. T13~T15: The leaf of ‘Xiaoye’; T16~T18: The leaf of ‘Qinzhong 2’. The color scale on the right represents the relative expression level based on FPKM values, where red and blue bars indicate high and low expression, respectively. Blue represents negative correlation, while red represents positive correlation. The solid line indicates that the correlation results reached a statistically significant level; the dashed line indicates that the correlation results did not reach a significant level.

Previous studies have identified at least 52 genes in *E. ulmoides* involved in the biosynthesis of *E. ulmoides* rubber (Holton et al., 1993). To explore the potential roles of *EuFLS* genes in *E. ulmoides* rubber biosynthesis, we analyzed their expression patterns using the transcriptomic data from leaves of two *E. ulmoides* cultivars: ‘Qinzhong 2’ (high *E. ulmoides* rubber content) and ‘Xiaoye’ (low *E. ulmoides* rubber content). The results are shown in **Figure 7**. The FPKM values of *EuFLS7*, *EuFLS8*, and *EuFLS9* were zero in both cultivars, indicating that these genes were not expressed in the leaves of either variety. In addition, the expression levels of eight *EuFLSs* were relatively low, with FPKM values < 10. In the ‘Xiaoye’ cultivar, the expression level of *EuFLS2* was relatively high, suggesting *EuFLS2* might play a negative regulatory role in the formation of *E. ulmoides* rubber. Conversely, in the ‘Qinzhong 2’ cultivar, the expression levels of *EuFLS6* and *EuFLS10* were significantly high, indicating that the two genes potentially play a positive regulatory role in the formation of *E. ulmoides* rubber.

To clarify the potential association between EuFLS genes and genes involved in the *E. ulmoides* rubber biosynthesis pathway in E. ulmoides, we constructed a co-expression network encompassing 12 EuFLS genes and two key *E. ulmoides* rubber biosynthesis genes—EuHMGR (3-hydroxy-3-methylglutaryl-CoA reductase) and EuFPS (farnesyl pyrophosphate synthase) (**Figure 7B**). The screening thresholds were |r| ≥0.60 and p < 0.05. The analysis results showed that there was a highly significant positive correlation between *EuFLSs* and these core *E. ulmoides* rubber biosynthesis genes. In the positive regulatory co-expression network, we identified 10 pairs correlated between 3 *E. ulmoides* rubber structural genes and 6 *EuFLS* genes. Statistical analysis showed that four *EuFLS* genes (*EuFLS2*, *EuFLS3*, *EuFLS6* and *EuFLS10*) had a degree of connection 2, suggesting that these four genes may play crucial positive regulatory roles in *E. ulmoides* rubber biosynthesis, though their specific functions require further experimental validation.

### 3.6 Analysis of the roles of *EuFLSs* in leaf color formation

**Figure 8:**
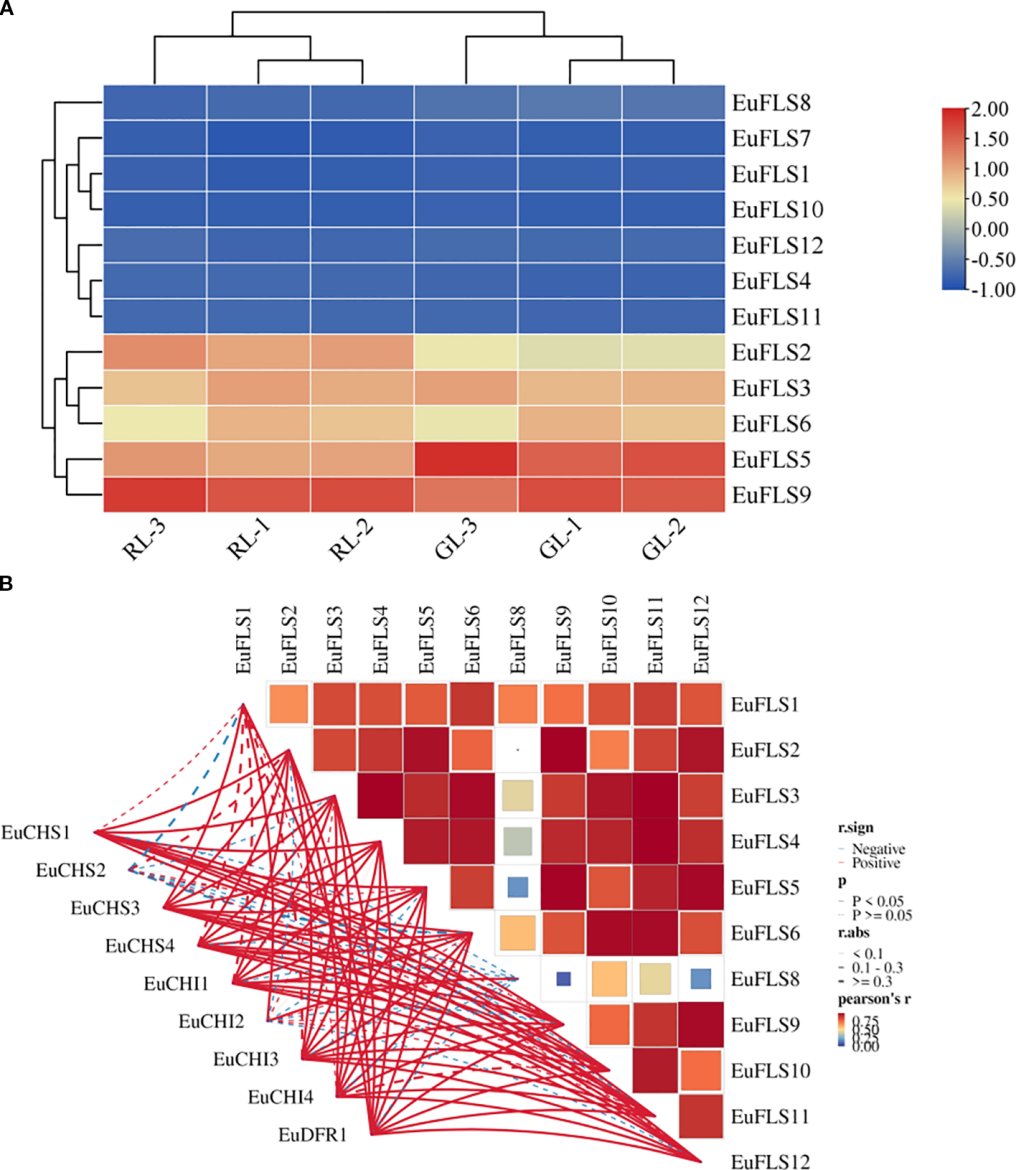
Expression patterns of *EuFLS* genes in different colored leaves of *E. ulmoides* **(A)**. and correlation analyses between EuFLS genes and EuCHS, EuCHI, EuDFR genes **(B)**. RL1~RL3: Red-leaf *E. ulmoides*; GL1~GL3: Green leaves *E. ulmoides*. The color key on the right represents the relative expression level in FPKM values, the red and blue bars (FPKM) indicate high and low expression, respectively. Blue represents negative correlation, while red represents positive correlation. The solid line indicates that the correlation results reached a statistically significant level; the dashed line indicates that the correlation results did not reach a significant level.

FLSs play an important role in the formation of leaf color, flower color, and fruit color. To analyze the function of *FLSs* in the formation of leaf color in *E. ulmoides*, we assessed their expression patterns using transcriptomic data from young and old leaves of both green-leaf and purple-leaf *E. ulmoides* varieties. The results were shown in **Figure 8**. The FPKM value of *EuFLS7* was zero in both varieties, indicating that it was not expressed in either variety. Among the 12 *EuFLS* genes, 10 *EuFLSs* were highly expressed in the red-leaf *E. ulmoides*, while only *EuFLS8* showed a slightly higher expression level in the green-leaf *E. ulmoides* than in the red-leaf. This was consistent with the expression patterns in other species, indicating that *EuFLS* is involved in flavonoid synthesis in *E. ulmoides*, and the specific functions need to be further verified.

To clarify the potential association between *EuFLS* genes and those involved in the flavonoid synthesis pathway in *E. ulmoides*, we constructed a co-expression network encompassing 12 *EuFLS* genes and three key flavonoid synthesis genes—*EuCHI* (Chalcone isomerase), *EuCHS* (Chalcone synthase), and *EuDFR* (Dihydroflavone 4-reductase) (**Figure 8B**). The screening thresholds were set at |r| ≥ 0.80 and p < 0.01. Analysis results revealed a highly significant positive correlation between *EuFLSs* and these core flavonoid synthesis genes. Within the positive regulatory co-expression network, 39 correlated pairs were identified between the 3 flavonoid synthesis structural genes and 9 *EuFLS* genes. Statistical analysis showed that *EuFLS11* had a correlation degree of 4, while five *EuFLS* genes (*EuFLS2*, *EuFLS4*, *EuFLS5*, *EuFLS9*, and *EuFLS12*) each had a connection degree of 6. These findings suggest that these five genes may play crucial roles in positively regulating flavonoid biosynthesis, though their specific functions require further experimental validation.

### 3.7 Analysis of expression patterns of *EuFLSs* in different tissues and flower development periods

**Figure 9:**
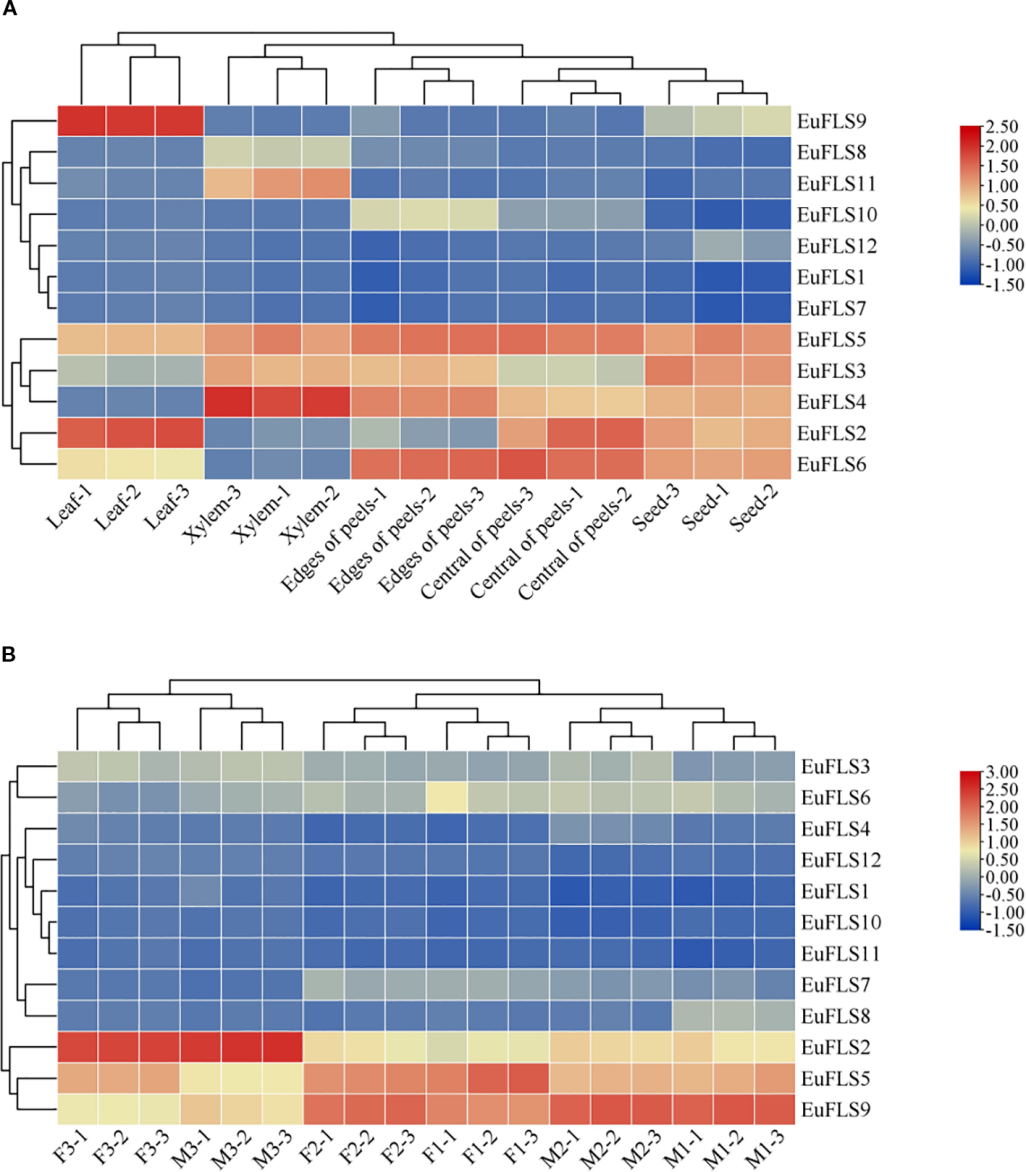
Expression patterns of *EuFLS* genes in different tissues **(A)** and flower development **(B)**. The color key on the right represents the relative expression level in FPKM values, the red and blue bars (FPKM) indicate high and low expression, respectively.

To comprehensively investigate the expressions of *EuFLSs* across different tissues, a heatmap was constructed based on transcriptomic data from various tissues of *E. ulmoides*, including leaves, seeds, marginal pericarp, central pericarp, and xylem. As illustrated in **Figure 9**, the FPKM values of *EuFLS3*, *EuFLS7*, and *EuFLS10* were zero in the leaves, indicating that these genes are not functionally active in this tissue. The transcription levels of *EuFLS2* and *EuFLS9* exhibited a distinct pattern, reaching their highest in the leaves and lowest in the xylem. This finding suggested that these genes primarily function in the leaves, with minimal activity in the xylem. Moreover, *EuFLS4* levels were the highest in the xylem. *EuFLS1* and *EuFLS7* were hardly expressed in all tissues. Additionally, *EuFLS2*, *EuFLS6*, *EuFLS9* and *EuFLS12* levels were the lowest in xylem. Collectively, these results demonstrated that *EuFLSs* exhibit tissue-specific expression patterns and play distinct functional roles in the development of different tissues in *E. ulmoides*.

To comprehensively analyze the functions of *EuFLSs* in the flowers of *E. ulmoides*, which is a dioecious plant, we examined their expression patterns using transcriptomic data from different flower developmental stages. These stages included the initial stage of female floral organ morphological differentiation (F1), female floral organ induction stage (F2), female flower organ maturity stage (F3), the initial stage of male floral organ morphological differentiation (M1), male floral organ induction stage (M2), and male flower organ maturity stage (M3). Our results revealed that *EuFLS10* was minimally expressed across all stages of flower development. Similarly, *EuFLS11* was not expressed in flower maturity stage. As the floral organs developed, *EuFLS2* levels gradually increased, reaching its peak during the M3 and F3, and the average value of FPKM was 82.7, with significantly higher levels in male flowers than in female flowers. In contrast, the expression levels of *EuFLS8* gradually declined during floral development, with male flowers exhibiting significantly lower than female flowers, the transcriptional level was the lowest in the M3 with the average value of FPKM was 1.8. In male flowers, *EuFLS5* levels decreased progressively as floral development. The transcription levels of *EuFLS5* and *EuFLS7* peaked during F2, however *EuFLS8*, and *EuFLS12* peaked during M2. *EuFLS16* level was the highest during F1. On the contrary, the levels of *EuFLS2* was the lowest in F1 with the average value of FPKM was 11.86. Collectively, these findings demonstrated that *EuFLSs* exhibited functional differentiation during the flower development process of *E. ulmoides*.

### 3.8 qRT-PCR detection of the expression patterns of *EuFLSs* in different tissues

**Figure 10:**
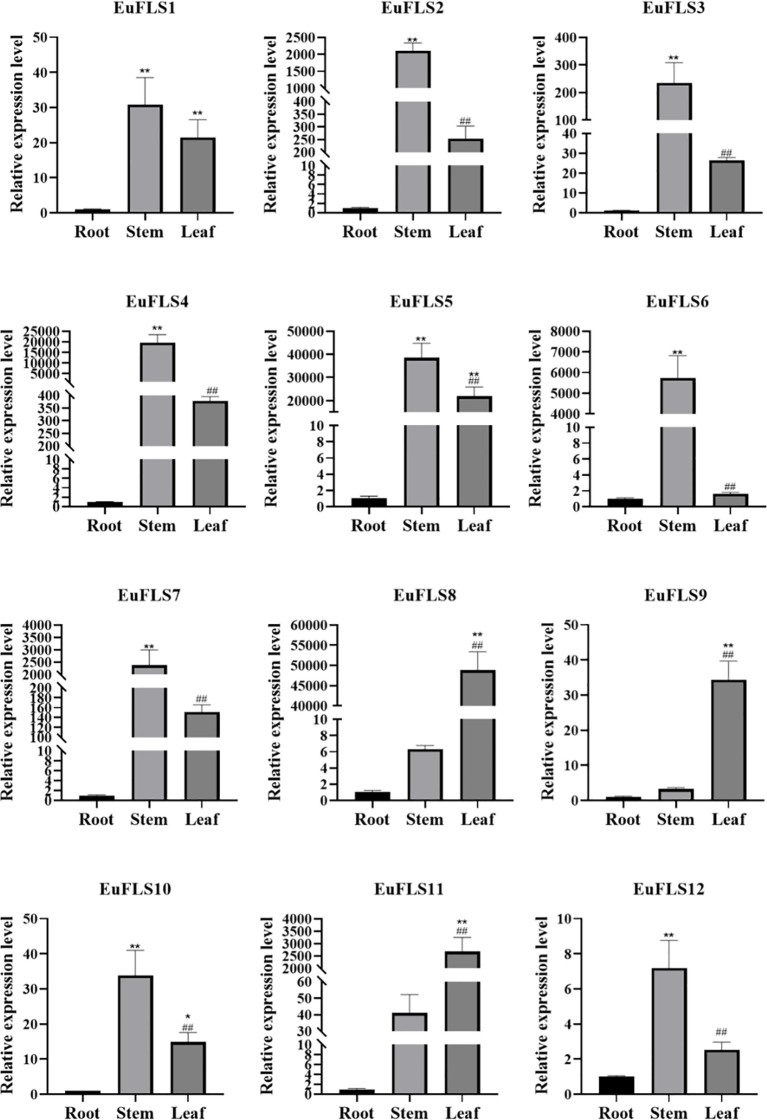
Expression pattern of *EuFLS* genes in different tissues by qRT-PCR. qRT-PCR data were normalized using *UBC* as the reference gene and were displayed relative to 0 h. Results were expressed as mean ± standard deviation (SD). Error bars represented the standard error of the mean of three independent replicates. The * indicated significant differences compared to the roots, as determined by t-test (*p<0.05, **p<0.01), the # indicated significant differences compared to the stems, as determined by t-test (^#^p<0.05, ^##^p<0.01).

To thoroughly investigate the functional roles of *EuFLSs* in various tissues of *E. ulmoides*, we selected roots, stems, and leaves from 2-month-old seedlings as experimental material and quantified the expression levels of *EuFLSs* in these tissues using qRT-PCR. The results revealed that the expression levels of *EuFLS2/3/4/5/6/7/10* were the highest in the stem (**Figure 10**). *EuFLS1/8/9/11/12* were highly expressed in the leaves. Notably, the levels of all *EuFLSs* were lowest in the roots. These findings demonstrated that *EuFLSs* exhibited remarkable tissue specificity, with distinct expression patterns across different tissue types.

### 3.9 Analysis of the expression patterns of the *EuFLSs* under hormone treatments

**Figure 11:**
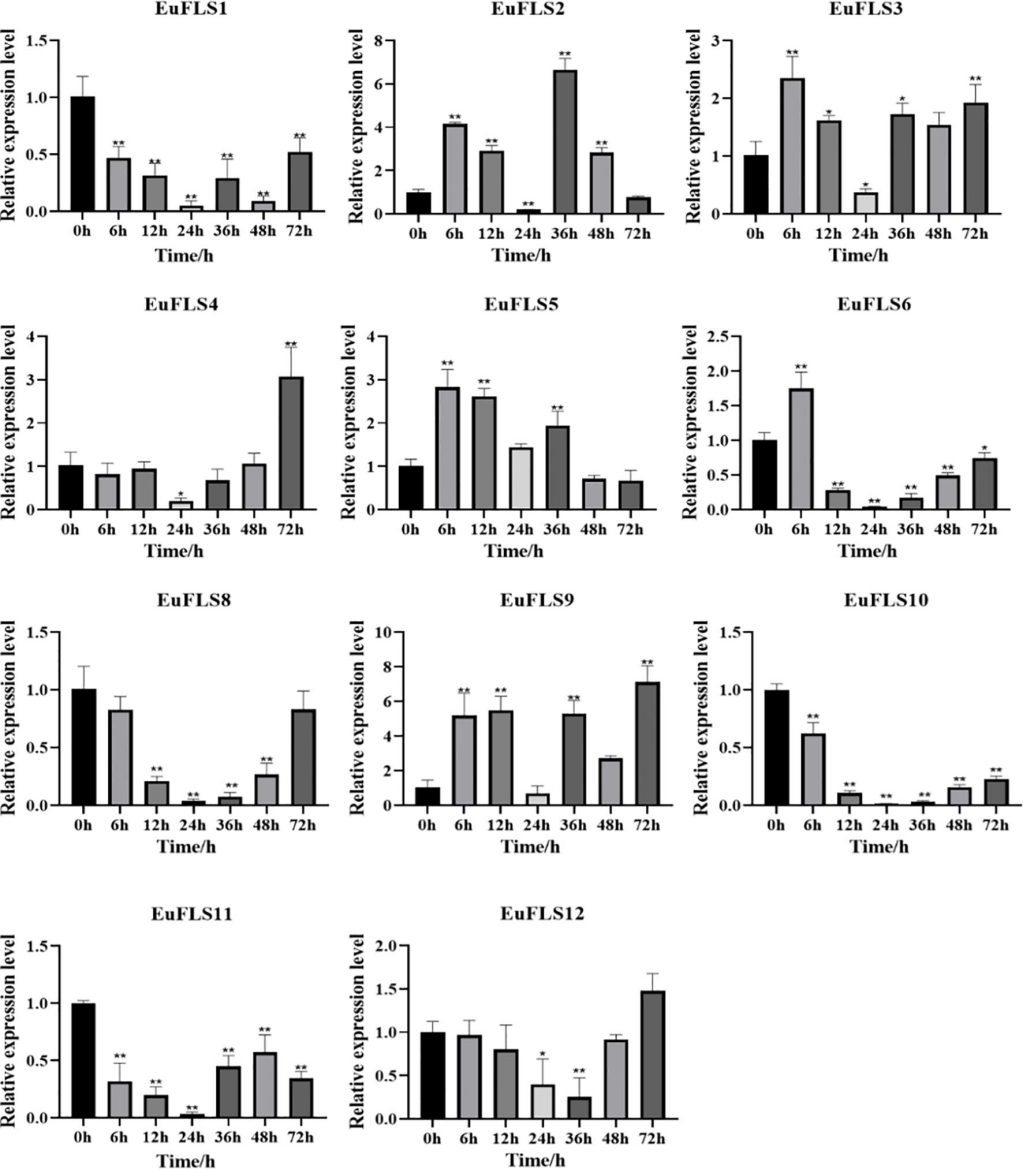
Expression patterns of *EuFLS* genes under ABA treatment. The horizontal axis represented ABA treatments, and the vertical axis represented relative gene expression levels. qRT-PCR data were normalized using *UBC* as the reference gene and were displayed relative to 0 h. Results were expressed as mean ± standard deviation (SD). Error bars represented the standard error of the mean of three independent replicates, the * indicated significant differences by t-test (*p<0.05, **p<0.01).

To explore the potential functions of *EuFLSs* in abiotic stress responses, we subjected *E. ulmoides* seedlings to hormone treatments with exogenous ABA, GA_3_, SA, and MeJA treatments, and systematically analyzed the expression patterns of *EuFLSs* using qRT-PCR. Under ABA treatment, *EuFLSs* exhibited significantly distinct expression trends, indicating that they might play varying regulatory roles in the ABA signaling pathway (**Figure 11**). More specifically, *EuFLS1*/*4*/*8*/*10*/*11*/*/12* consistently downregulated under ABA stress, exhibiting the lowest levels at 24 h post-treatment. Among them, *EuFLS10* exhibited the most significant sustained inhibitory effect, with its expression in the treated group being only 1/66 times its expression in the control group. In addition, the levels of *EuFLS8* and *EuFLS11* decreased to 1/33 times the levels in the control group, while *EuFLS1* levels decreased to 1/25 times the levels in the control group. In contrast, *EuFLS4* and *EuFLS12* displayed biphasic expression patterns, characterized by an initial decline followed by a significant upregulation, peaking at 72 h post-treatment. Notably, *EuFLS5* and *EuFLS6* exhibited a transient induction response, with their transcript levels rapidly peaking at 6 h post-treatment, reaching 2.6 and 1.7 times their levels in the control group, respectively, before gradually declining to baseline levels. Furthermore, *EuFLS3* and *EuFLS9* exhibited oscillatory expression patterns. *EuFLS3* displayed an early-phase induction, reaching its first peak (2.3-fold higher levels than in control) at 6 h post-treatment. In contrast, *EuFLS9* showed delayed upregulation, achieving maximum expression (7.3-fold higher levels than in control) at 72 h post-treatment. Of particular significance was the expression dynamics of *EuFLS2*, which showed a polyphasic trend. Its expression peaked at 36 h post-treatment (6.7-fold higher levels than in control) and then fluctuated subsequently. These differential expression patterns suggested that *EuFLSs* might participate in ABA-mediated stress responses.

**Figure 12:**
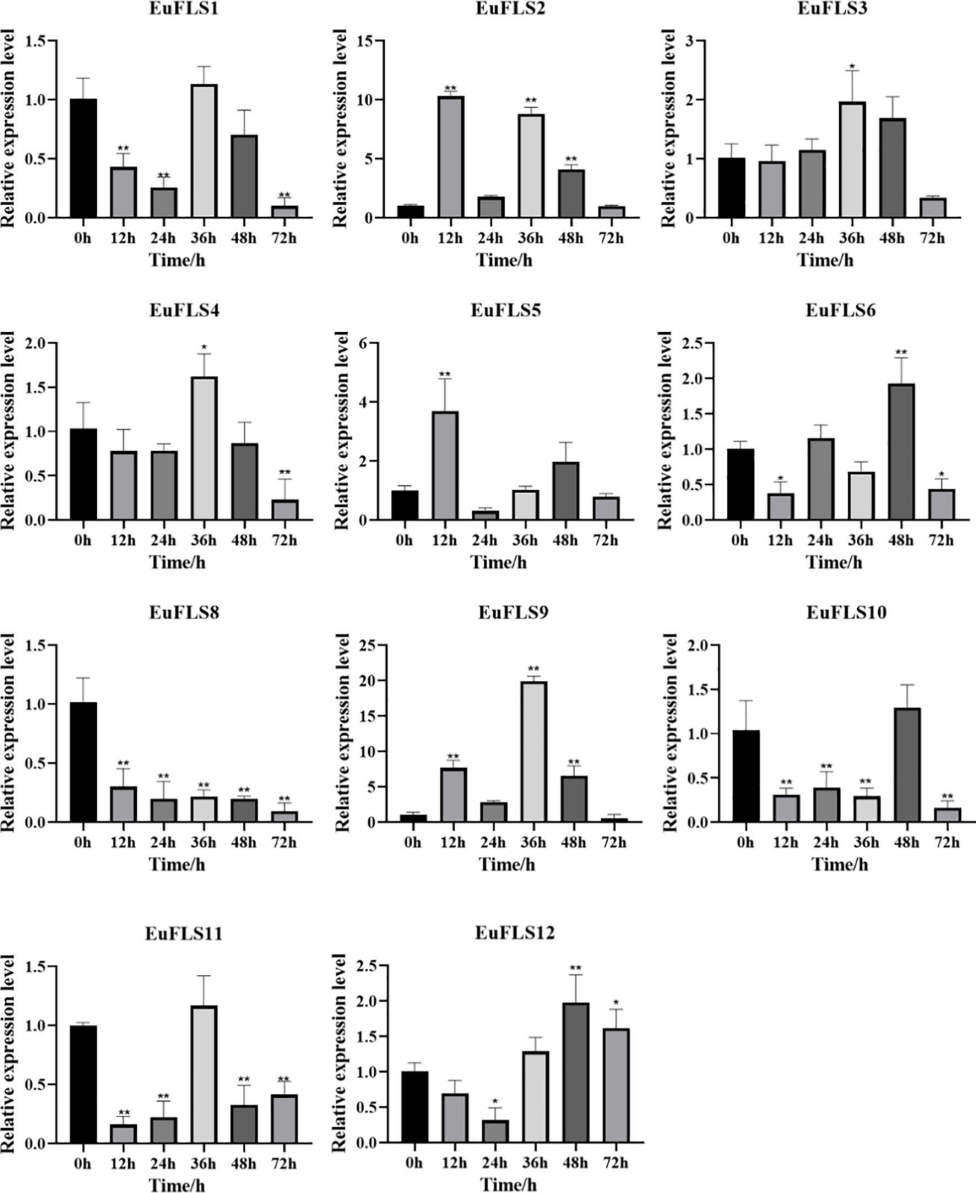
Expression patterns of *EuFLS* genes under GA_3_ treatment. The horizontal axis represented GA_3_ treatments, and the vertical axis represented relative gene expression levels. 0 h was selected as the control group. qRT-PCR data were normalized using *UBC* as the reference gene and were displayed relative to 0 h. Results were expressed as mean ± standard deviation (SD). Error bars represented the standard error of the mean of three independent replicates, the * indicated significant differences by t-test (*p<0.05, **p<0.01).

Under GA_3_ treatment, *EuFLSs* exhibited diverse dynamic expression patterns (**Figure 12**). The levels of *EuFLS1*/*3*/*4*/*6*/*10*/*11* first decreased, then increased, and finally decreased. Among them, the expression level of *EuFLS1*/*3*/*4*/*11* peaked at 36 h post-treatment, with the most prominent upregulation observed for *EuFLS3* (2-fold higher expression than in control). In contrast, the expression levels of *EuFLS6* and *EuFLS10* reached their maximum at 48 h after treatment, with the treated group showing a significantly higher (1.9-fold increase) *EuFLS6* expression compared to the control group. *EuFLS5* and *EuFLS9* displayed rapidly responsive upward expression trends. *EuFLS5* peaked at 12 h (3.7-fold higher expression than in control) and then declined rapidly. Although *EuFLS9* showed a delayed response (36 h), it exhibited a strong induction effect, with the treated group exhibiting 20-fold higher *EuFLS9* levels than the control group. However, *EuFLS8* exhibited consistently downward expression, suggesting that the GA_3_ stress inhibits its expression. *EuFLS12* displayed a biphasic trend characterized by an initial suppression followed by activation and peaking at 48 h, with a 1.9-fold increase in expression levels. *EuFLS2* displayed the most complex expression dynamics, presenting a wavy pattern. Its expression level is particularly high at 12 h (10-fold higher expression than in control). This finding implied that *EuFLS* genes might play multiple regulatory roles at different stages of GA_3_ signal transduction.

**Figure 13:**
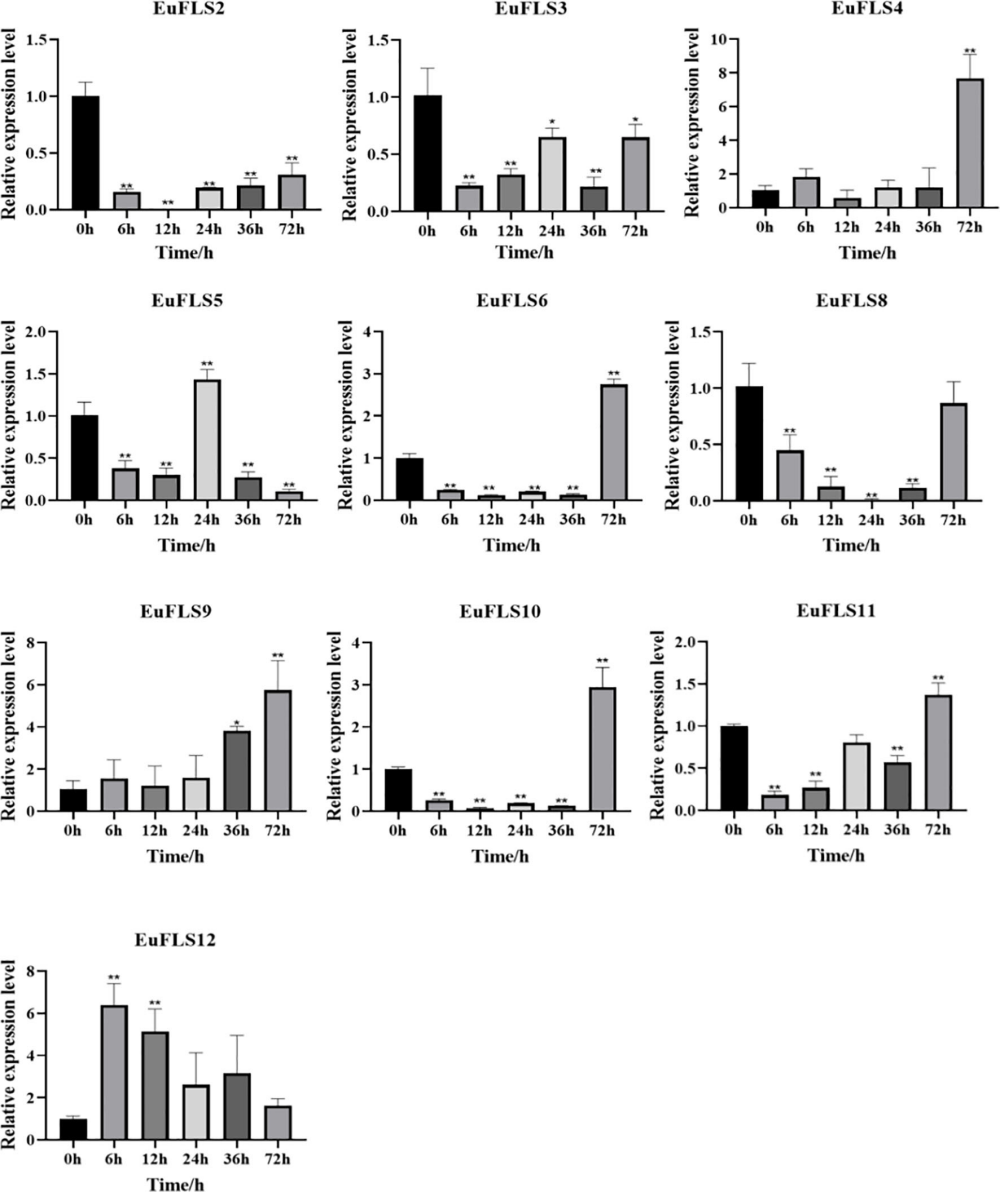
The expression profile of *EuFLS* genes under SA treatment. The horizontal axis represented SA treatments, and the vertical axis represented relative gene expression levels. 0 h was selected as the control group. qRT-PCR data were normalized using *UBC* as the reference gene and were displayed relative to 0 h. Results were expressed as mean ± standard deviation (SD). Error bars represented the standard error of the mean of three independent replicates, the * indicated significant differences by t-test (*p<0.05, **p<0.01).

Similarly, under SA treatment, *EuFLSs* exhibited significantly differential expression patterns (**Figure 13**), specifically manifested as *EuFLS6*/*10*/*11* displayed similar expression dynamics, characterized by an initial downregulation, followed by upregulation, all peaking at 72 h post-treatment with 2.7-, 3-, and 1.4-fold increases in expression, respectively. On the contrary, *EuFLS2*/*3*/*8* exhibited sustained downregulation throughout the treatment. *EuFLS4* exhibited more complex regulatory characteristics. Its expression showed a fluctuating trend by first increasing, then decreasing, and then increasing again. Finally, its levels peaked at 72 h post-treatment, being 7-fold higher than those in control. However, *EuFLS5* exhibited an oscillating expression pattern by first decreasing, then increasing, and then decreasing again. Its levels peaked at 24 h post-treatment, being 1.5-fold higher than those in control. Furthermore, both *EuFLS9* and *EuFLS12* were consistently upregulated, peaking at 72 and 6 h post-treatment, respectively (6- and 6.2-fold higher expressions than in control, respectively). These varied expression patterns suggested that during SA-mediated stress, *EuFLSs* might play critical physiological roles via temporal regulation.

**Figure 14:**
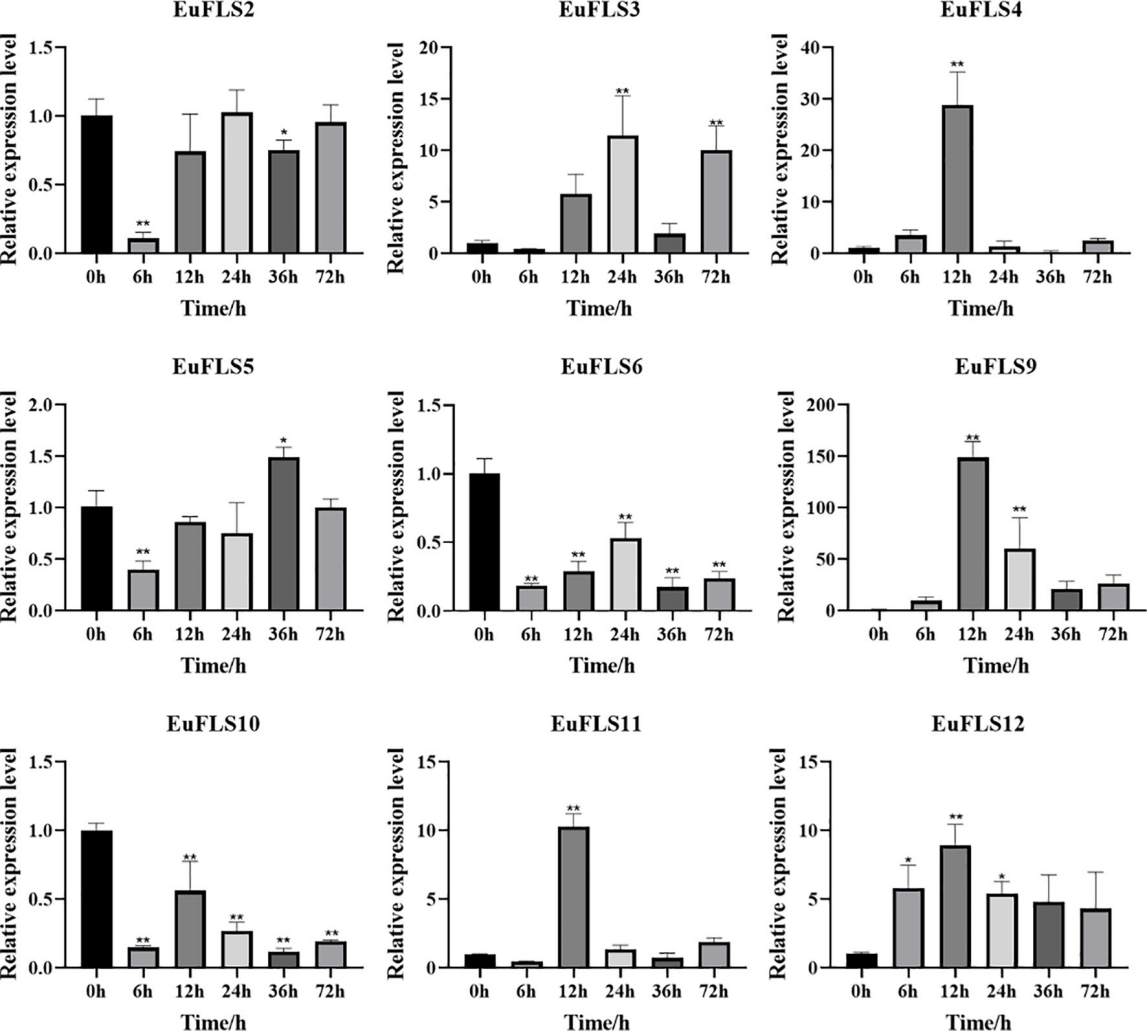
Expression patterns of *EuFLS* genes during the treatment with MeJA. The horizontal axis represented MeJA treatments, and the vertical axis represented relative gene expression levels. 0 h was selected as the control group. qRT-PCR data were normalized using *UBC* as the reference gene and were displayed relative to 0 h. Results were expressed as mean ± standard deviation (SD). Error bars represented the standard error of the mean of three independent replicates, the * indicated significant differences by t-test (*p<0.05, **p<0.01).

Under MeJA treatment (**Figure 14**), both *EuFLS2* and *EuFLS5* showed fluctuating expressions by first decreasing, then increasing, and then decreasing again. Among them, *EuFLS5* levels peaked at 36 h post-treatment, reaching 1.5 times the levels in the control group. However, *EuFLS9* and *EuFLS12* exhibited a consistent upregulation, both peaking at 12 h post-treatment, with levels increasing by 150- and 8.3-fold than the levels in the control group, respectively. This finding indicated that these two genes might be involved in the early defense response against MeJA. *EuFLS3* levels initially decreased and then increased, peaking at 24 h post-treatment (11-fold higher expression than in control). Notably, *EuFLS4* and *EuFLS11* exhibited more complex regulatory patterns. *EuFLS4* levels first increased, then decreased, and then increased again, peaking at 12 h post-treatment (28-fold higher expression than in control). The expression level of *EuFLS11* exhibited a multiphase change pattern by first decreasing, then increasing, then decreasing, and finally increasing again. Its levels also peaked at 12 h post-treatment (10-fold higher expression than in control). In contrast, *EuFLS6* and *EuFLS10* were consistently downregulated, indicating that they were negatively regulated under MeJA stress.

### 3.10 The expression patterns of *EuFLSs* under drought stress

**Figure 15:**
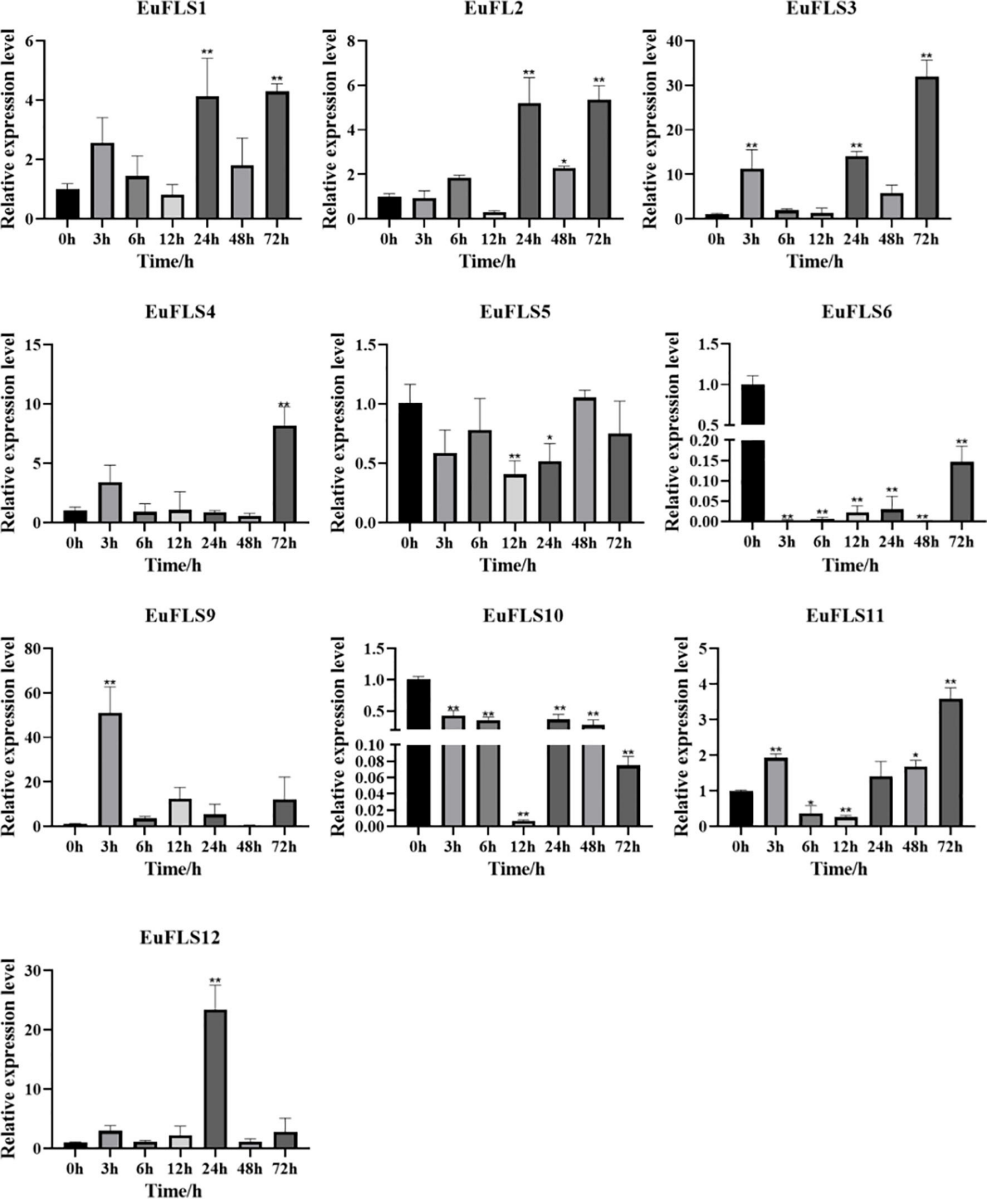
Expression patterns of *EuFLS* genes under the treatment with PEG6000. The horizontal axis represented PEG6000 treatments, and the vertical axis represented relative gene expression levels. 0 h was selected as the control group. qRT-PCR data were normalized using *UBC* as the reference gene and were displayed relative to 0 h. Results were expressed as mean ± standard deviation (SD). Error bars represented the standard error of the mean of three independent replicates, the * indicated significant differences by t-test (*p<0.05, **p<0.01).

Under drought stress, *EuFLSs* exhibited significantly divergent spatiotemporal expression patterns, which could be classified into four types based on their expression characteristics (**Figure 15**). First was the sustained downregulation type. *EuFLS6* and *EuFLS10* levels consistently decreased throughout the drought stress, indicating that these genes might be inhibited by drought stress. Second was the biphasic oscillation type. *EuFLS5* exhibited a unique “inhibition-induction-fallback” expression pattern, suggesting that this gene might be involved in the regulation of stage-specific responses to drought stress. Third was the multimodal response type. *EuFLS1*/*2*/*3*/*4*/*9*/*11*/*12* showed complex “induction-inhibition-reinduction” expression patterns. Among them, *EuFLS9* exhibited the fastest response, with the highest expression level at 3 h post-treatment (50-fold higher than in control). Furthermore, *EuFLS12* and *EuFLS3* levels peaked at 24 and 72 h, respectively (22- and 30-fold higher expressions than in control, respectively). Such differential expression patterns implied functional divergence among *EuFLSs* under drought stress responses. The pronounced upregulation of these specific *EuFLS* genes indicates that they were key components of the *E. ulmoides* molecular response to drought stress. Their potential role in drought tolerance remains to be functionally characterized, but their expression patterns make them interesting candidates for further investigation.

## 4 Discussion

*E. ulmoides* is an ancient and precious plant that has been used as medicine in China for more than 2000 years. Because its bark, leaves, seeds, and male flowers can be used in medicine, it plays an important role in medicine, food, chemical industry, and other fields, so it is also called “plant gold” (Bao et al., 2024). Flavonoids, one of the major bioactive constituents of *E. ulmoides*, are widely distributed and abundant in this species. FLS, a key enzyme in the flavonoid biosynthesis pathway, specifically catalyzes the 3-hydroxylation of dihydroflavonols to produce flavonols. To date, *FLSs* have been identified and extensively studied in various plant species, including petunia (Holton et al., 1993), *Arabidopsis* (Owens et al., 2008), tobacco (Huang et al., 2006), *M. armeniacum* (Xu et al., 2011), *Fagopyrum tataricum* (Sun, 2012), tea plant (Ma and Chen, 2009), *Malus domestica* (Liu et al., 2018), *Myrica rubra* (Xing, 2021), and *Triticum aestivum* (Qi, 2016). However, *FLSs* in *E. ulmoides* remain uncharacterized, highlighting the need for further research on their functional roles in flavonoid biosynthesis in this species. In this study, twelve members of the EuFLS gene family (designated *EuFLS1*-*EuFLS12*) were identified in *E. ulmoides*. The analysis of physicochemical properties reveals a high variation among the different members. The variation range of the amino acid sequence of EuFLSs was 328 aa to 420 aa, with molecular weights ranging from 36297.19 Da to 46513.98 Da, and the expected the oretical isoelectric point ranging from 5.46 to 8.72. In terms of gene structure, the range of variation in *EuFLS* genes exons ranged from 3 to 5, and all *EuFLS* genes harbored introns. Among other species, intron variation in maize flavonoid-related gene synthesis ranged from 0 to 11, with 9.71% lacking introns (Ge et al., 2021). In rice, the number of introns in *FLS* genes ranged from 0 to 10, with 5.88% of these genes being intron-free (Park et al., 2019). On the contrary, intron counts in tomato *FLS* genes varied from 1 to 11, and nearly all tomato *FLS* genes contained introns (Wei et al., 2021). Based on the gene structure analysis of alfalfa, the intron number in *MsFLS* genes spanned from 2 to 6, and all *MsFLS* genes harbored introns (Yue et al., 2024). Given that differences in gene structure exert a significant impact on gene function, we speculate that the FLS gene family in E. ulmoides has fewer introns and low genetic diversity. This characteristic may help reduce gene redundancy and enhance functional specificity of EuFLS genes. A total of 12 *EuFLS* genes were unevenly distributed across eight chromosomes (**Figure 4**), which differs from the AthFLS gene family in Arabidopsis—a family primarily localized near chromosome 5 (Owens et al., 2008). Phylogenetic tree analysis revealed that most *EuFLS* genes within the same clade shared similar exon-intron organization patterns and conserved motifs, despite variations in intron length among some members (**Figure 3**). This phenomenon suggests that the intron organization of homologous genes tends to be conserved during evolution, which is consistent with findings reported in alfalfa (Yue et al., 2024). Collectively, these differences not only provide a basis for the classification of the FLS gene family but also indicate that FLS family members may have undergone functional differentiation.

The distribution pattern of cis-acting elements within promoters can offer insights into their functional roles in plant signal transduction pathways and the regulation of complex biological processes. In this study, a total of 270 cis-acting elements were identified, harboring a wide variety of cis-regulatory elements related to hormone signaling and stress adaptation. The main types of these elements are those involved in auxins, jasmonic acid, SA, ABA, and drought response (**Figure 5**). Among these, light-responsive elements were the most abundant (n=137), followed by hormone- (n=91) and abiotic stress-responsive elements (n=72). This distribution suggested that *EuFLSs* are involved in hormone signaling, abiotic stress and photoperiod regulation in *E. ulmoides*. Previous studies show that ABA induces the expression of flavonoid synthesis-related genes, enhancing stress resistance in pigeon peas (Yang et al., 2021). In tobacco, GA3, ABA, jasmonic acid (JA), MeJA, and SA treatments induced *NtFLS1* expression, while drought and high-temperature stresses significantly upregulated both *NtFLS1* and *NtFLS2* in tobacco roots (He et al., 2024). In alfalfa, *MsFLSs* respond to cold stress to varying degrees, overexpression of *MsFLS13* increases flavonoid levels and reduces accumulation of ROS, Malondialdehyde (MDA), CO_2_, and H_2_O_2_ under cold stress (Yue et al., 2024). In cotton, promoters of *GhFLS* mainly contain plant hormone response elements and abiotic stress-responsive elements, notably, *GhFLS* genes expression is induced by salt, drought, low temperature, and heat stress, while heterologous expression of *GhFLS1* reduces salt stress tolerance (Han et al., 2023). These findings further confirm that *EuFLSs* may play a significant role in both hormonal responses and abiotic stress responses. However, the specific function of individual *EuFLSs* genes still need to be verified through functional validation approaches, such as gene overexpression and gene knockout.

The expression of *FLS* genes exhibits distinct tissue-specific and developmental stage-specific patterns across plant species. For example, in *Solanum sisymbriifolium*, *SsFLS2* shows the highest expression level in roots, followed by leaves and stems (Sun et al., 2024). In *M. domestica*, *MdFLS2* is expressed in multiple tissues including roots, stems, leaves, flowers, and young fruits, with its expression peaking in leaves (Qi et al., 2018). In Allium cepa, *FLS* genes are highly expressed in leaf sheaths, moderately expressed in leaves, and nearly undetectable in roots (Wang et al., 2022). Aligning with the aforementioned results, *EuFLS* genes also display distinct tissue- and stage-specific expression. *EuFLS8*/*9*/*11* were highly expressed in leaves, while *EuFLS1/2/3/4/5/6/7*/*10* and *EuFLS12* were mainly expressed in stems, Interestingly, the expression of all *EuFLS* genes was lowest in roots. For comparison, in other plant species: In *Juglans regia*, *JrFLS1*/*4*/*5*/*7* exhibited high expression in vegetative buds, pistils, and mature leaves, and *JrFLS1* and *JrFLS5* were primarily expressed at the early stage of female flower bud differentiation (Zhang et al., 2025). In *Vitis vinifera*, five *VvFLSs* were expressed in flower buds and flowers, *VvFLS4* and *VvFLS5* were detectable in mature fruits, and *VvFLS4* expression was dependent on light (Fujita et al., 2006). In *Citrus unshiu*, *CitFLS* was more highly expressed in immature fruits compared to mature fruits (Moriguchi et al., 2002). In *Eriobotrya japonica*, during fruit ripening, *FLS* expression has been shown to be positively correlated with flavonol accumulation while negatively correlated with dihydroflavonol levels (Zheng, 2019). Previous studies show that the accumulation of most flavonoids peaks in growing leaves, follow by old leaves of *E. ulmoides* by metabolic analysis, concurrently, the expression levels of most isoforms of *F3’H*, *FLS*, *CHI* and *CHS* are relatively high in growing leaves, resulting in high concentrations of naringenin chalcone, aromadendrin, kaempferol and quercetin in the growing leaves stage (Li et al., 2019). Consistent with these previous findings, the result of the present study show that *EuFLS2/3/5/6/10* are highly expressed in both growing leaves and old leaves, further supporting the notion that these genes play a significant role in flavonoid synthesis.

In the flavonoid biosynthesis pathway, *FLS* competes with *DFR* for substrates, forming a key regulatory node that directs metabolic flux toward either anthocyanin or flavonol production, thereby influencing plant pigmentation and leaf coloration (Luo et al., 2016). For *Chrysanthemum morifolium*, studies have demonstrated that overexpression CmFLS in tobacco results in elevated flavone/flavonol content and reduced anthocyanin levels, with expression levels of *CmFLS* being positively associated with flower color intensity (Wang et al., 2021). In *Ipomoea batatas*, *IbFLS1* exhibits expression in young green leaves. When IbFLS1 was silenced, the plant developed a purple phenotype; concurrently, the genes DFR, ANS, and UFGT were upregulated, ultimately causing an increase in anthocyanin accumulation and a decrease in flavonol accumulation (Xiao et al., 2024). In this study, expression profiling of *EuFLSs* in differently colored leaves revealed distinct expression patterns. Only *EuFLS8* was highly expressed in green-leafed, whereas the remaining *EuFLS* genes all showed the higher expression level in purple leaves, suggesting that *EuFLS* is involved in *E. ulmoides* leaf color formation. In *Camellia nitidissima*, *CnFLS1* expression has been found to positively correlate with flavonol accumulation. Heterologous overexpression of *CnFLS1* in tobacco resulted in flower color transition to white or pale yellow, demonstrating that *CnFLS1* negatively regulates yellow pigment deposition (Zhou et al., 2013). In *Petunia hybrida*, antisense suppression of *FLS* expression led to inhibition of flavonol biosynthesis in petals, causing a phenotypic shift from light pink to red coloration (Holton et al., 1993; Nielsen et al., 2002). In sweet potato, silencing of the *FLS* gene leads to purple leaf coloration, along with the up-regulated expression of *DFR*, *ANS* and *UFGT*. This genetic modification also results in a significant increase in total anthocyanin content, while total flavonol content decreases remarkably (Xiao et al., 2024). Based on this evidence, we speculate that *EuFLSs* not only contribute to flavonol synthesis but also serve as potential candidate genes involved in leaf color formation in *E. ulmoides*.

Flavonols are known to be involved in the development of floral organs and flower fertility (Mo et al., 1992; Napoli et al., 1999). For instance, knockout of *CHS* gene in petunias and silencing of *FLS* gene in tobacco can both inhibit anther whitening, induce male sterility, and influence other related traits (Napoli et al., 1999; Mahajan et al., 2011). In *Camellia sinensis*, the content of flavonols correlates with stamen growth, most flavonol glycosides are primarily accumulated in petals and anthers, and *CsFLSb* exhibits high expression in anthers in fertile flowers while being expressed at extremely low levels in sterile flowers (Shi et al., 2021). Additionally, in tobacco, adding low-concentration flavonols to the germination medium can restore pollen germination and pollen tube elongation (Ylstra et al., 1992). In our study, *EuFLS9* showed the highest expression level during the induction stage of both female and male flowers of E. ulmoides and was also highly expressed in purple leaves. These results imply that *EuFLS9* may play an important role in mediating the involvement of flavonols in the formation and development of E. ulmoides floral organs, though further in-depth mechanistic research is needed to confirm this.

Plant hormones are widely recognized to play pivotal roles in regulating plant growth, development, and responses to diverse environmental stresses. Furthermore, these phytohormones intricately regulate the biosynthesis of secondary metabolites, notably flavonols, within plants. Previous studies have shown that the *FLS* family plays an important role in the regulating various stresses, such as ABA, SA, MeJA, and drought stress. In *tartary buckwheat*, the expression of *FtFLS1* is suppressed by SA and ABA, whereas *FtFLS2* is unaffected by ABA but induced by SA (Li et al., 2013). In *Camellia vietnamensis* Huang, *FLS* genes—potential regulators of flavonoid formation—exhibited elevated expression 2 h following MeJA treatment (Yan et al., 2022). In this study, the expression levels of *EuFLS2/3/5/6/9* genes were enhanced during the early stage of ABA treatment. The induction effect of GA_3_ treatment on *EuFLSs* was even more pronounced, upregulating eight *EuFLS* genes. *EuFLS2* levels increased by 10-fold at 12 h post-treatment, and *EuFLS9* levels increased by 20-fold at 36 h. Following SA treatment, six *EuFLSs* were significantly upregulated, with *EuFLS4* being the most responsive, showing a 7-fold increase at 72 h post-treatment compared to the control. These findings were consistent with the expression patterns of tobacco *FLSs* (*NtFLSs*), ABA and SA have been found to markedly upregulate *NtFLS1* and *NtFLS2*, additionally, *NtFLS1* is also upregulated by GA_3_, with its levels increasing by 2.07-fold (He et al., 2024). In *Dendrobium officinale*, SA treatment significantly upregulated *DoFLS1* expression at 2 h post-treatment, with 4.66-fold higher levels than in control (Yu et al., 2021). Previous studies have revealed that *FLS* gene in leaves exhibits peak expression levels in plants treated with MeJA and SA during the vegetative growth stage (Sun et al., 2012; Pandey et al., 2016). In *Euphorbia kansui*, *EkFLS* expression peaked its maximum at 12–36 h post-MeJA treatment, and this induction was associated with increased flavonoid accumulation in both leaves and roots. Additionally, when *EkFLS* was expressed in Arabidopsis, it improved the plant’s tolerance to drought and salt stress while increasing the contents of total flavonoids and flavonols (Zhang, 2020). In the study, six *EuFLSs* showed significant upregulation under MeJA treatment, with the expression level of E*uFLS9* increasing by 150-fold at 12 h post-treatment. Notably, *EuFLS9* was also induced to express under the treatments of ABA, GA3 and MeJA, therefore, we assumed that *EuFLS9* directly participate in the ABA -GA_3_-MeJA crosstalk in *E. ulmoides*.

Studies have shown that under salt, drought stress and MeJA treatments, plant flavonoid content increases significantly, which helps enhance tolerance to abiotic stresses (Azhar et al., 2011; Li et al., 2020a). In *Arabidopsis*, overexpression of *EkFLS* increased the content of total flavonoid, quercetin and kaempferol under the stresses of MeJA, NaCl and PEG, which can inhibit ABA-induced stomatal closure, improving tolerance to drought and salt stress (Wang et al., 2021b). In *Castanea mollissima*, drought stress upregulated the expression of CmFLS; when CmFLS was heterologously overexpressed in tobacco, it enhanced the plant’s drought tolerance by increasing antioxidant enzyme activity and promoting flavonol accumulation (Zeng, 2024). In *M. domestica*, *MdFLS1* expression has been found to be induced by high salinity, drought, low temperature, and ABA treatment. Additionally, overexpression of *MdFLS1* improved salt and drought tolerance in apple callus (Liu, 2018). Similarly, in *Carthamus tinctorius*, the expression of *CtFLS1* showed a positive correlation with flavonoid content, *CtFLS1* overexpression in *Arabidopsis* not only promoted seed germination, improve osmotic and drought stress tolerance, and reduced ABA sensitivity, furthermore, it also upregulated key flavonol biosynthetic genes while downregulating anthocyanin synthesis genes. These results suggest that *CtFLS1* alleviates drought sensitivity by stimulating flavonol and anthocyanin accumulation (Zhang et al., 2025). In the study, the expression of most *EuFLS* genes was also regulated to varying degrees under drought treatment. Notably, *EuFLS3* was significantly upregulated, with its expression level increasing by 30-fold at 72 h post-treatment; *EuFLS9*, by contrast, responded a strong early response to drought, peaking at 3 h post-treatment with an expression level 50-fold higher than that of the control. These results suggest that *EuFLS3* and *EuFLS9* may play crucial roles in promoting flavonoids biosynthesis, thereby enhancing the drought stress tolerance of *E. ulmoides.* Future research using transgenic approaches will be essential to validate whether the manipulation *EuFLS3* and *EuFLS9* genes can indeed confer enhanced drought tolerance, thus confirming their potential as breeding targets. Concurrently *EuFLS3* and *EuFLS9* can be used as a candidate marker to accelerate the selection of drought-tolerant seedlings, reducing the time cost associated with traditional breeding. Furthermore, science flavonoids act as antioxidants to alleviate drought-induced oxidative damage, targeted overexpression of stress-responsive *EuFLS* genes may simultaneously enhance drought tolerance and maintain stable rubber yield—providing a new strategy for biotechnological improvement of *E. ulmoides.*

Above all, *EuFLSs* genes were induced by multiple treatments such as ABA, GA_3_, SA, MeJA, as well as by drought stress. Previous studies have shown that phytohormone crosstalk is universally present in plants, constituting a very complex signaling regulatory network that plays a critical role in the regulation of growth and development in plants (Lian et al., 2024). In our study, we postulate that *FLS* genes in *E. ulmoides* respond to various stresses through different hormone signaling pathways, such as ABA-GA_3_ stress crosstalk, SA-MeJA crosstalk, or drought stress crosstalk, regulating the growth and development of *E. ulmoides*. For future research, investigating the functional of *EuFLS* genes in regulating growth and development, adversity stress response, and hormone crosstalk regulation must include transgenic assays. These assays will be essential to definitively validate the proposed connections between *EuFLS* function, flavonoid metabolism, and rubber biosynthesis in E. ulmoides.

## 5 Conclusion

In this study, we conducted a comprehensive and systematic characterization of the flavonol synthase (FLS) gene family in *E. ulmoides*. A total of 12 *EuFLS* genes were identified, and their physicochemical properties, chromosomal localization, conserved domain, gene structures, promoter cis-elements, and phylogenetic relationships were analyzed. The results showed that 12 *EuFLSs* genes could be classified into three main subgroups of I and III, and members within the same subgroup had similar motif compositions and gene structures. Additionally, we analyzed the expression profiles of *EuFLSs* genes in different tissues, distinct leaf developmental stages, *E. ulmoides* cultivars with varying rubber contents, different leaf colors, and different developmental stages of male and female flowers, as well as their expression responses to drought stress and various hormonal treatments. Collectively, the extensive bioinformatics and expression analysis of *EuFLSs* genes contributes to our understanding of the functions of these genes in multiple stress responses and phytohormone crosstalk.

## Data Availability

The datasets presented in this study can be found in online repositories. The names of the repository/repositories and accession number(s) can be found in the article/**Supplementary Material**.
