# Supplementary material for: Genome-wide identification and comprehensive analysis of EuFLS genes in Eucommia ulmoides reveals their roles in growth, development, and abiotic stress response
Source: Front Plant Sci. 2025 Oct 23;16:1662635. doi: 10.3389/fpls.2025.1662635 (PMC12589077; doi:10.3389/fpls.2025.1662635)
Supplement: Supplementary file 2 [file Table1.doc]

Table S1. The FPKM of the *EuFLSs* in *E. ulmoides* leaves at different developmental stages

| Gene name | T01 | T02 | T03 | T04 | T05 | T06 | T07 | T08 | T09 | T10 | T11 | T12 |
| --- | --- | --- | --- | --- | --- | --- | --- | --- | --- | --- | --- | --- |
| *EuFLS1* | 0.09 | 0.10 | 0.09 | 0.09 | 0.08 | 0.07 | 0.11 | 0.10 | 0.11 | 0.11 | 0.11 | 0.10 |
| *EuFLS2* | 42.58 | 37.98 | 43.93 | 516.50 | 489.56 | 464.12 | 10.98 | 11.86 | 11.27 | 40.69 | 35.57 | 40.00 |
| *EuFLS3* | 20.40 | 19.08 | 20.16 | 32.04 | 27.83 | 29.36 | 3.31 | 3.08 | 3.01 | 1.08 | 1.13 | 1.52 |
| *EuFLS4* | 0.00 | 0.00 | 0.00 | 0.00 | 0.00 | 0.00 | 0.00 | 0.00 | 0.00 | 0.00 | 0.00 | 0.00 |
| *EuFLS5* | 1.49 | 1.87 | 2.03 | 4.09 | 3.76 | 4.20 | 0.63 | 1.07 | 0.90 | 0.60 | 0.80 | 0.72 |
| *EuFLS6* | 0.41 | 0.60 | 2.83 | 4.87 | 5.75 | 1.19 | 62.79 | 57.59 | 57.13 | 117.95 | 98.92 | 109.85 |
| *EuFLS7* | 0.00 | 0.00 | 0.00 | 0.00 | 0.00 | 0.00 | 0.00 | 0.00 | 0.00 | 0.00 | 0.00 | 0.00 |
| *EuFLS8* | 0.00 | 0.00 | 0.00 | 0.00 | 0.00 | 0.00 | 0.00 | 0.00 | 0.00 | 0.00 | 0.00 | 0.00 |
| *EuFLS9* | 0.00 | 0.00 | 0.00 | 0.00 | 0.00 | 0.00 | 0.00 | 0.00 | 0.00 | 0.00 | 0.00 | 0.00 |
| *EuFLS10* | 4.76 | 3.61 | 3.38 | 4.72 | 7.49 | 4.70 | 63.01 | 61.10 | 63.86 | 114.52 | 97.56 | 125.90 |
| *EuFLS11* | 6.86 | 8.76 | 9.11 | 1.07 | 1.33 | 1.60 | 0.81 | 1.08 | 0.97 | 1.01 | 1.10 | 1.09 |
| *EuFLS12* | 0.47 | 0.50 | 0.82 | 0.44 | 0.21 | 0.27 | 0.07 | 0.76 | 0.07 | 0.02 | 0.18 | 0.00 |

Table S2 The FPKM of *E.ulmoide EuFLS*s in leaves of different gutta percha

| Gene name | T13 | T14 | T15 | T16 | T17 | T18 |
| --- | --- | --- | --- | --- | --- | --- |
| *EuFLS1* | 0.10 | 0.11 | 0.08 | 0.19 | 0.09 | 0.09 |
| *EuFLS2* | 32.24 | 28.39 | 37.75 | 5.27 | 4.53 | 6.92 |
| *EuFLS3* | 32.24 | 28.39 | 37.75 | 5.27 | 4.53 | 6.92 |
| *EuFLS4* | 0.00 | 0.00 | 0.00 | 0.00 | 0.00 | 0.00 |
| *EuFLS5* | 0.74 | 0.90 | 0.99 | 0.53 | 1.41 | 0.49 |
| *EuFLS6* | 4.39 | 13.74 | 12.08 | 35.03 | 40.25 | 50.01 |
| *EuFLS7* | 0.00 | 0.00 | 0.00 | 0.00 | 0.00 | 0.00 |
| *EuFLS8* | 0.00 | 0.00 | 0.00 | 0.00 | 0.00 | 0.00 |
| *EuFLS9* | 0.00 | 0.00 | 0.00 | 0.00 | 0.00 | 0.00 |
| *EuFLS10* | 8.46 | 13.89 | 9.63 | 28.33 | 34.65 | 47.68 |
| *EuFLS11* | 11.66 | 14.19 | 15.12 | 20.26 | 18.04 | 21.50 |
| *EuFLS12* | 0.12 | 0.03 | 0.22 | 0.17 | 0.07 | 0.75 |

*Table S3 FPKM of the EuFLS genes in different leaf colors of E. ulmoides*

| Gene name | GL-1 | GL-2 | GL-3 | RL-1 | RL-2 | RL-3 |
| --- | --- | --- | --- | --- | --- | --- |
| *EuFLS1* | 0.06 | 0.03 | 0 | 0.1 | 0.07 | 0.04 |
| *EuFLS2* | 5.7 | 4.97 | 4.24 | 89.97 | 84.45 | 78.92 |
| *EuFLS3* | 13.93 | 12.09 | 10.25 | 101.49 | 67.11 | 32.72 |
| *EuFLS4* | 0.08 | 0.07 | 0.06 | 0.49 | 0.35 | 0.21 |
| *EuFLS5* | 40.71 | 36.92 | 33.12 | 84.97 | 76.13 | 67.29 |
| *EuFLS6* | 15.02 | 9.57 | 4.12 | 71.50 | 43.76 | 16.02 |
| *EuFLS7* | 0 | 0 | 0 | 0 | 0 | 0 |
| *EuFLS8* | 0.5 | 0.37 | 0.23 | 0.54 | 0.35 | 0.15 |
| *EuFLS9* | 50.93 | 33.48 | 16.02 | 384.20 | 341.96 | 299.71 |
| *EuFLS10* | 0 | 0 | 0 | 0.15 | 0.08 | 0 |
| *EuFLS11* | 0.13 | 0.11 | 0.08 | 0.5 | 0.36 | 0.21 |
| *EuFLS12* | 0.2 | 0.17 | 0.13 | 0.36 | 0.34 | 0.31 |

*Table S4 FPKM of the EuFLSs in different tissues of E. ulmoides*

| Gene name | Leaf-1 | Leaf-2 | Leaf-3 | Seed-1 | Seed-2 | Seed-3 | Edges of peels-1 | Edges of peels-2 | Edges of peels-3 | Central of peels-1 | Central of peels-2 | Central of peels-3 | Xylem-1 | Xylem-2 | Xylem-3 |
| --- | --- | --- | --- | --- | --- | --- | --- | --- | --- | --- | --- | --- | --- | --- | --- |
| *EuFLS1* | 0 | 0 | 0 | 0 | 0 | 0 | 0 | 0 | 0 | 0 | 0 | 0 | 0.05 | 0.04 | 0 |
| *EuFLS2* | 240.01 | 235.85 | 223.80 | 32.72 | 31.08 | 27.17 | 3.89 | 1.38 | 0.86 | 71.79 | 70.51 | 19.23 | 1.01 | 0.78 | 0.20 |
| *EuFLS3* | 4.72 | 2.58 | 2.23 | 55.54 | 43.4 | 41.14 | 19.27 | 17.69 | 13.16 | 5.62 | 3.83 | 3.68 | 21.98 | 19.45 | 19.23 |
| *EuFLS4* | 0.19 | 0.14 | 0 | 43.26 | 30.72 | 19.48 | 42.79 | 30.26 | 29.95 | 16.66 | 13.97 | 13.12 | 117.88 | 109.94 | 101.71 |
| *EuFLS5* | 37 | 33.04 | 28.75 | 77.87 | 45.07 | 25.50 | 47.65 | 42.18 | 40.85 | 48.85 | 46.79 | 38.26 | 52.97 | 24.78 | 22.37 |
| *EuFLS6* | 18.82 | 13.4 | 10.15 | 46.85 | 38.64 | 27.46 | 54.85 | 47.97 | 47.66 | 63.45 | 60.82 | 53.43 | 0.64 | 0.38 | 0.10 |
| *EuFLS7* | 0 | 0 | 0 | 0 | 0 | 0 | 0 | 0 | 0 | 0.05 | 0 | 0 | 0 | 0 | 0 |
| *EuFLS8* | 0.21 | 0.16 | 0 | 0.55 | 0.35 | 0.34 | 1.35 | 0.67 | 0.42 | 0.40 | 0.2 | 0.07 | 4.54 | 4.16 | 3.69 |
| *EuFLS9* | 579.17 | 390.12 | 336.38 | 8.54 | 8.58 | 3.52 | 1.79 | 0.28 | 0.07 | 0.48 | 0.07 | 0.02 | 0.19 | 0.15 | 0.09 |
| *EuFLS10* | 0 | 0 | 0 | 0.09 | 0.07 | 0 | 6.87 | 5.75 | 4.78 | 1.68 | 1.24 | 1.05 | 0.15 | 0.11 | 0 |
| *EuFLS11* | 0.54 | 0.16 | 0.04 | 0.91 | 0.68 | 0 | 0.49 | 0.37 | 0 | 0.45 | 0.34 | 0 | 35.34 | 32.65 | 13.51 |
| *EuFLS12* | 0.17 | 0.1 | 0.03 | 4.09 | 2.02 | 0.51 | 0.10 | 0.06 | 0.02 | 0.22 | 0.16 | 0.04 | 0.05 | 0.04 | 0 |

*Table S5 FPKM of the EuFLSs in different flower development of E. ulmoides*

| Gene name | F1-1 | F1-2 | F1-3 | M2-1 | M2-2 | M2-3 | M1-1 | M1-2 | M1-3 | F2-1 | F2-2 | F2-3 | F3-1 | F3-2 | F3-3 | M3-1 | M3-2 | M3-3 |
| --- | --- | --- | --- | --- | --- | --- | --- | --- | --- | --- | --- | --- | --- | --- | --- | --- | --- | --- |
| *EuFLS1* | 0.07 | 0.05 | 0 | 0.11 | 0.08 | 0 | 0 | 0 | 0 | 0.1 | 0.05 | 0 | 0 | 0 | 0 | 0.84 | 0.03 | 0.10 |
| *EuFLS2* | 14.24 | 11.93 | 9.49 | 64.75 | 40.54 | 21.86 | 42.37 | 21.53 | 16.21 | 39.36 | 28.51 | 18.27 | 132.45 | 130.79 | 98.94 | 301.70 | 250.70 | 245.60 |
| *EuFLS3* | 4.24 | 2.36 | 2.10 | 10.68 | 6.35 | 6.05 | 2.86 | 2.74 | 2.22 | 5.47 | 4.53 | 3.35 | 4.48 | 3.9 | 2.13 | 4.58 | 4.41 | 4.04 |
| *EuFLS4* | 0.15 | 0.13 | 0.09 | 3.67 | 2.5 | 1.38 | 1.04 | 0.79 | 0.56 | 0.12 | 0.1 | 0.07 | 0.72 | 0.29 | 0.10 | 0.31 | 0.23 | 0.13 |
| *EuFLS5* | 148.22 | 126.91 | 108.58 | 91.18 | 77.38 | 43.59 | 60.63 | 58.2 | 56.03 | 160.66 | 147.32 | 143.80 | 29.17 | 26.2 | 22.69 | 15.45 | 11.59 | 10.99 |
| *EuFLS6* | 23.21 | 6.06 | 4.54 | 15.82 | 9.77 | 7.01 | 10.98 | 6.48 | 4.00 | 9.17 | 5.23 | 4.80 | 1.48 | 0.69 | 0.63 | 2.74 | 2.71 | 2.69 |
| *EuFLS7* | 4.83 | 3.25 | 2.14 | 4.87 | 2.66 | 2.25 | 2.33 | 2.28 | 0.81 | 6.65 | 3.87 | 3.85 | 0.22 | 0.05 | 0.0 | 0.05 | 0.01 | 0 |
| *EuFLS8* | 1.08 | 0.48 | 0.27 | 1.64 | 1.46 | 0.72 | 7.41 | 6.35 | 4.01 | 0.56 | 0.54 | 0.48 | 0.32 | 0.18 | 0.14 | 0.23 | 0.205 | 0.13 |
| *EuFLS9* | 138.86 | 62.46 | 44.45 | 473.49 | 420.68 | 182.27 | 267.30 | 253.39 | 164.56 | 290.57 | 280.94 | 269.15 | 9.66 | 8.81 | 6.84 | 27.19 | 16.97 | 12.56 |
| *EuFLS10* | 0.13 | 0.06 | 0.02 | 0.32 | 0.1 | 0.06 | 0.58 | 0.25 | 0.06 | 0.23 | 0.2 | 0.2 | 0.04 | 0.01 | 0 | 0.11 | 0.09 | 0.04 |
| *EuFLS11* | 0.18 | 0 | 0 | 0.85 | 0.48 | 0.20 | 0 | 0 | 0 | 0.37 | 0.01 | 0 | 0 | 0 | 0 | 0 | 0 | 0 |
| *EuFLS12* | 0.71 | 0.31 | 0.24 | 0.67 | 0.45 | 0.41 | 0.90 | 0.47 | 0.27 | 0.66 | 0.53 | 0.35 | 0.38 | 0.29 | 0.24 | 0.51 | 0.34 | 0.22 |
